# Environmentally Friendly Synthesis of New Mono- and Bis-Pyrazole Derivatives; In Vitro Antimicrobial, Antifungal, and Antioxidant Activity; and In Silico Studies: DFT, ADMETox, and Molecular Docking

**DOI:** 10.3390/ph18020167

**Published:** 2025-01-26

**Authors:** Oussama Merzouki, Nadia Arrousse, Elhachmia Ech-chihbi, Ashwag S. Alanazi, El Houssine Mabrouk, Mohamed Hefnawy, Abdelfattah El Moussaoui, Hanane Touijer, Azeddin El Barnossi, Mustapha Taleb

**Affiliations:** 1 Laboratory of Engineering Electrochemistry, Modeling, and Environment, Department of Chemistry, Faculty of Sciences Dhar Mahraz, Sidi Mohamed Ben Abdellah University, Fez 30000, Morocco; 2 School of Science and Engineering, Al Akhawayn University in Ifrane, Hassan II Avenue, Ifrane 53000, Morocco; 3 Laboratory of Physics and Chemistry of Inorganic and Organic Materials, Higher Normal School, Mohammed V University, Rabat 30050, Morocco; 4 Department of Pharmaceutical Sciences, College of Pharmacy, Princess Nourah Bint Abdulrahman University, Riyadh 11671, Saudi Arabia; 5 Laboratory of Materials Engineering for the Environment and Natural Ressources, Faculty of Sciences and Technics, University of Moulay Ismail, Meknes, B.P 509, Boutalamine, Errachidia 52000, Morocco; 6 Department of Pharmaceutical Chemistry, College of Pharmacy, King Saud University, Riyadh 11451, Saudi Arabia; 7 Plant Biotechnology Team, Faculty of Sciences, Abdelmalek Essaadi University, Tetouan 93002, Morocco; 8 Laboratory of Biotechnology, Environment, Agrifood, and Health, Faculty of Sciences Dhar El Mahraz, Sidi Mohamed Ben Abdellah University, Fez 30050, Morocco

**Keywords:** synthesis, pyrazole derivatives, medicinal activities, molecular docking, ADMET

## Abstract

**Background/Objectives:** Antimicrobial resistance and oxidative stress are major global health challenges, necessitating the development of novel therapeutic agents. Pyrazole derivatives, known for their diverse pharmacological properties, hold promise in addressing these issues. This study aimed to synthesize new mono- and bis-pyrazole derivatives using an eco-friendly, catalyst-free approach and evaluate their antioxidant, antibacterial, and antifungal activities, supported by in silico ADMET profiling, molecular docking, and Density Functional Theory (DFT) analysis. **Methods:** The compounds were synthesized via a green condensation reaction and characterized using NMR and mass spectrometry, which was verified by DFT analysis. Biological activities were assessed through DPPH and FRAP antioxidant assays, as well as disk diffusion and MIC methods, against bacterial strains (*Pseudomonas aeruginosa*, *Staphylococcus aureus*, and *Escherichia coli*) and fungal strains (*Candida albicans* and *Aspergillus niger*). Computational ADMET profiling evaluated pharmacokinetics and toxicity, while molecular docking assessed interactions with target proteins, including catalase, topoisomerase IV, and CYP51. **Results:** Theoretical calculations using DFT were in agreement with the experimental results; regarding biological activities, O4 demonstrated the most significant antioxidant activity, with 80.14% DPPH radical scavenging and an IC50 value of 40.91 µg/mL. It exhibited potent antimicrobial activity, surpassing Streptomycin with a 30 mm inhibition zone against Pseudomonas aeruginosa and showing strong efficacy against Staphylococcus aureus and Candida albicans. Computational studies confirmed favorable pharmacokinetic properties, no AMES toxicity, and strong binding affinities. DFT analysis revealed O4’s stability and reactivity, further validating its potential as a therapeutic candidate. **Conclusions:** This study identified and characterized novel pyrazole derivatives with promising biological and pharmacological properties. O4 emerged as the most potent compound, demonstrating strong antioxidant and antimicrobial activities alongside favorable computational profiles. These findings highlight the potential of the synthetized compounds for therapeutic development and underscore the value of integrating green synthesis with computational techniques in drug discovery.

## 1. Introduction

**Figure 1:**
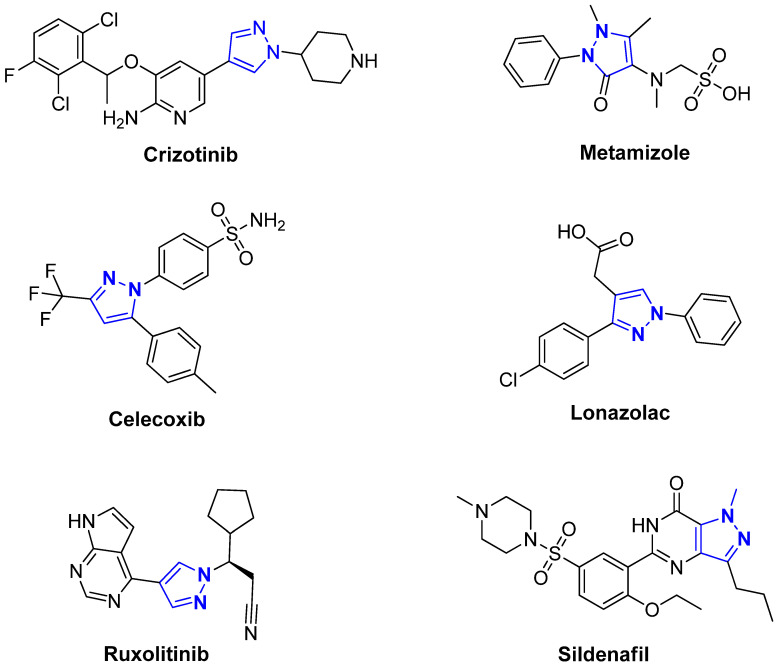
Drug compounds containing a pyrazole moiety in their structure.

Pyrazole, a five-membered heterocyclic compound with two adjacent nitrogen atoms, has emerged as a privileged scaffold in drug discovery due to its broad spectrum of biological activities [1,2,3,4]. This versatile structure is a core component of numerous clinically approved pharmaceuticals [5,6], including Crizotinib, Ruxolitinib, Tozasertib, Celecoxib, and Metamizole, demonstrating its adaptability across therapeutic categories, as shown in Figure 1. The inherent structural diversity of pyrazole allows for the design of derivatives with finely tuned pharmacological properties, making it a highly attractive target for medicinal chemistry [7,8,9,10]. Recent advances in synthetic methodologies have further facilitated the preparation of mono- and bis-pyrazole derivatives, enabling the exploration of their therapeutic potential in addressing critical health challenges [11,12].

The significance of mono- and bis-pyrazole derivatives lies in their unique structural properties, which influence their interactions with biological targets. Mono-pyrazoles, characterized by a single pyrazole ring, exhibit a variety of therapeutic activities, including antioxidant, anti-inflammatory, and anticancer properties [13,14,15,16]. In contrast, bis-pyrazole derivatives, containing two linked pyrazole moieties, often display enhanced biological activity due to synergistic effects and increased binding potential with target enzymes or receptors [17]. This duality in design not only broadens the scope of applications but also enables the development of highly potent compounds tailored to specific medical needs [18].

The rationale for synthesizing new mono- and bis-pyrazole derivatives is rooted in their potential to address two major global health concerns: antimicrobial resistance and oxidative stress [19,20,21]. The overuse and misuse of antibiotics have led to the emergence of multi-drug-resistant (MDR) bacterial strains, complicating the treatment of bacterial and fungal infections and contributing to elevated morbidity and mortality rates [22]. Furthermore, oxidative stress, resulting from an imbalance between antioxidant defenses and free radical production, exacerbates cellular damage and inflammation, intensifying the impact of infections and contributing to chronic conditions such as cardiovascular diseases, neurodegenerative disorders, and diabetes [23,24,25]. Designing compounds that can simultaneously mitigate oxidative damage and inhibit microbial growth represents a promising therapeutic strategy [26,27].

Computational techniques have become invaluable for exploring and developing therapeutically significant new drugs, as they reduce reliance on animal models and facilitate the systematic advancement of safe new drug candidates [28]. In the field of in silico molecular docking, analyses have clarified biological activities by identifying the stable orientations of synthesized chemical entities within the receptor pocket under examination. However, the clinical development process often necessitates the exclusion of multiple drug candidates, primarily due to pharmacokinetic issues, which can significantly affect both the cost and duration of the process. By integrating molecular docking and the ADMET method early in the drug discovery process, researchers can better predict potential pharmacokinetic challenges, thereby streamlining the selection of more viable candidates for clinical development [29].

In this context, the synthesis of novel mono- and bis-pyrazole derivatives offers a dual approach to addressing these challenges. The inclusion of functional groups within the pyrazole scaffold can enhance antioxidant properties through radical scavenging or ferric-reducing mechanisms, while structural modifications can improve antimicrobial activity by targeting key microbial enzymes. This study focuses on the green synthesis of mono- and bis-pyrazole derivatives using a catalyst-free approach, exploring their potential as therapeutic agents through experimental and computational analyses. By integrating Density Functional Theory (DFT), ADMET profiling, and molecular docking studies, the research aims to rationalize the structural features responsible for the observed biological activities and provide insights for future drug development.

## 2. Results and Discussion

### 2.1. General Synthesis of the Novel Compounds

**Figure 2:**
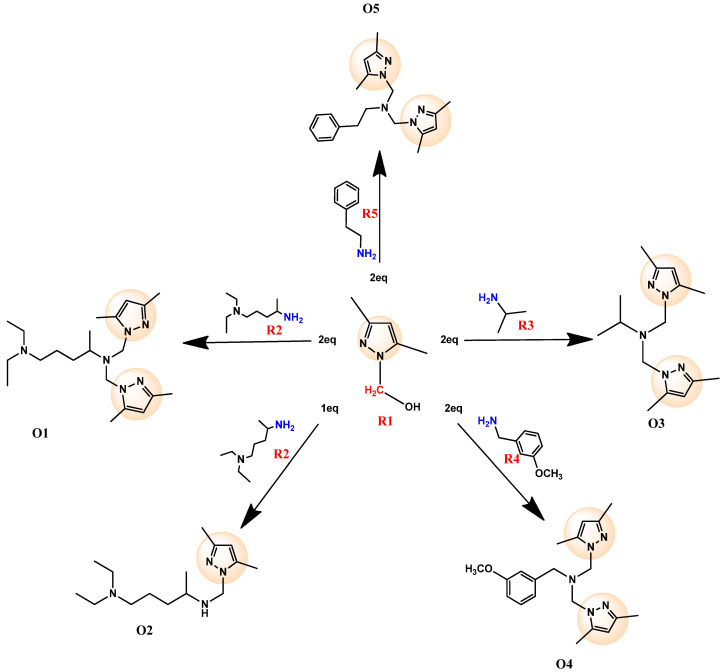
Synthesis of novel compounds containing bis- and mono-pyrazole moieties.

The synthesis of the novel pyrazole derivatives (O1–O5), illustrated in Figure 2, was carried out via a straightforward, eco-friendly method involving condensation reactions. The precursor, (3,5-dimethyl-1H-pyrazol-1-yl)methanol, was prepared by condensing 3,5-dimethylpyrazole with formaldehyde in ethanol under mild conditions, achieving yields above 80% [17]. The synthesized precursor was then reacted with various amines (diethylpentane-1,4-diamine, propan-2-amine, 3-methoxybenzylamine, and 2-phenylethanamine) in the presence of acetonitrile as a solvent. The reactions proceeded smoothly at moderate temperatures (55–60 °C) without requiring catalysts.

The resulting products were purified and characterized using spectroscopic techniques, including 1H NMR, 13C NMR, and mass spectrometry, confirming their structures and the successful introduction of functional groups. The yields of the synthesized compounds ranged from 61% to 77%, which, while moderate, are considered acceptable given the simplicity of the reaction conditions and the absence of any catalyst or toxic reagents. The yield range further underscores the practicality and reproducibility of the method under eco-friendly conditions.

This green, catalyst-free synthesis, as detailed in Figure 2, demonstrates an efficient and sustainable approach to producing mono- and bis-pyrazole derivatives. Despite moderate yields, the methodology provides a balance between environmental compatibility and structural diversity, enabling the exploration of the pharmacological potential of the resulting compounds. This approach not only minimizes environmental impact but also streamlines the synthetic process, making it a valuable contribution to sustainable drug discovery.

### 2.2. Global Reactivity Analysis Using the DFT Method

**Figure 3:**
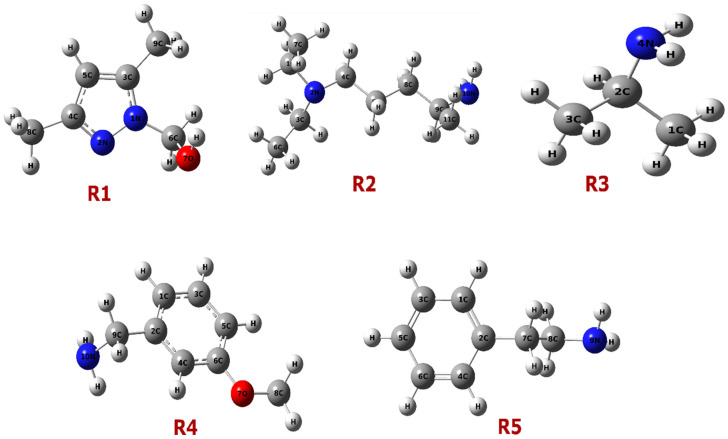
Optimized geometries of compounds R1, R2, R3, R4, and R5.

**Figure 4:**
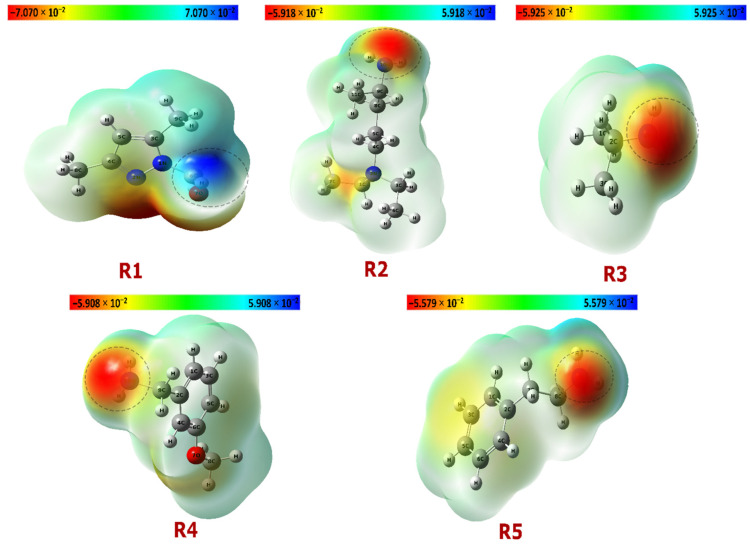
Molecular Electrostatic Potential (MEP) maps of compounds R1, R2, R3, R4, and R5.

The optimized geometries of reagents R(1–5) are displayed in Figure 3. The MEP maps shown in Figure 4 reveal the electron density distribution across the molecules, with color gradients indicating regions of high (red) and low (blue) electron density. Red regions denote electron-rich areas, while blue regions indicate electron-poor areas [30]. For instance, in R1, the electron-rich region around oxygen suggests its higher electronegativity, confirming its potential role in intermolecular interaction.

**Figure 5:**
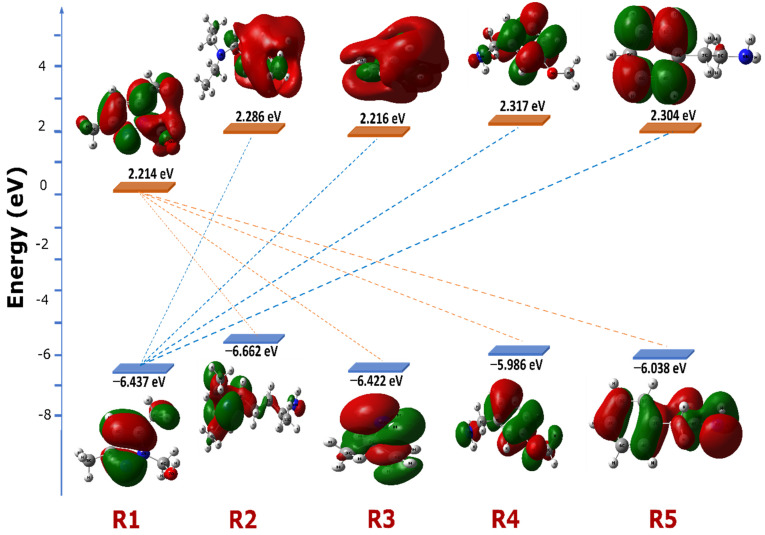
Energy diagram of the Highest Occupied Molecular Orbitals (HOMOs) and Lowest Unoccupied Molecular Orbitals (LUMOs) for the compounds R1, R2, R3, R4, and R5.

In Figure 5, the energy diagram illustrates the relative energy levels of the Highest Occupied Molecular Orbitals (HOMOs) and Lowest Unoccupied Molecular Orbitals (LUMOs) for the compounds R1, R2, R3, R4, and R5. These frontier molecular orbitals, or FMOs, are essential to understanding each molecule’s reactivity and stability. The diagram highlights the energy differences between the HOMO and LUMO for each compound, known as the “HOMO-LUMO gap”, which plays a crucial role in predicting chemical reactivity. Typically, a smaller HOMO-LUMO gap indicates a more reactive compound, as it requires less energy for electron transition. The HOMO and LUMO energies of TCE are −8.749 eV and −4.536 eV, respectively.

Table 1 and Figure 5 further detail these interactions, showing that the energy difference ∣E_HOMO_ (R_i_) − E_LUMO_ (R1)∣ is smaller than ∣E_HOMO_ (R1) − E_LUMO_ (R_i_)∣, suggesting that the most favorable HOMO-LUMO interaction involves the smallest energy gap. This relationship indicates that R1 preferentially interacts with the other compounds (R2, R3, R4, and R5), making reactions between R1 and these molecules more probable. Additionally, R1 exhibits electrophilic behavior, while the other molecules act as nucleophiles, favoring electron donation to R1. The analysis of chemical reactivity parameters in Table 1 supports these observations. The chemical potential (μ) values for R2 (μ = −1.688 eV), R3 (μ = −2.103 eV), R4 (μ = −1.834 eV), and R5 (μ = −1.867 eV) are higher than that of R1 (μ = −2.111 eV). This hierarchy in energy levels reinforces that R1 is more electrophilic compared to the other compounds, which have higher chemical potentials and therefore display nucleophilic characteristics. These findings are validated by comparing the global electrophilicity index (ω) values of the selected molecules, which are consistently lower than that of R1. The hardness (η) of R1 (η = 8.651 eV) is higher than that of R2, R3, R4, and R5, which have respective values of 7.948 eV, 8.638 eV, 8.303 eV, and 8.342 eV. This means that R1 maintains weak electrons in its environment; therefore, the electrons pass from R2, R3, R4, and R5 to R1.

### 2.3. Local Reactivity: Fukui Indices

**Figure 6:**
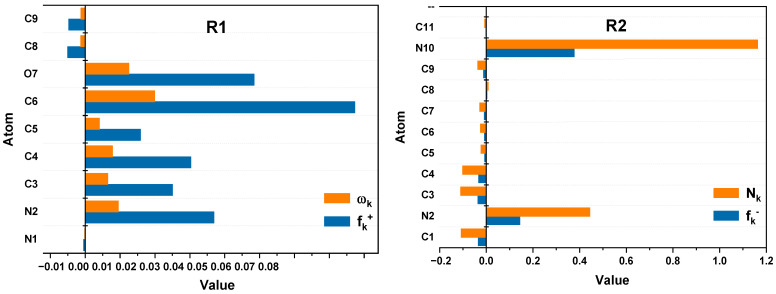

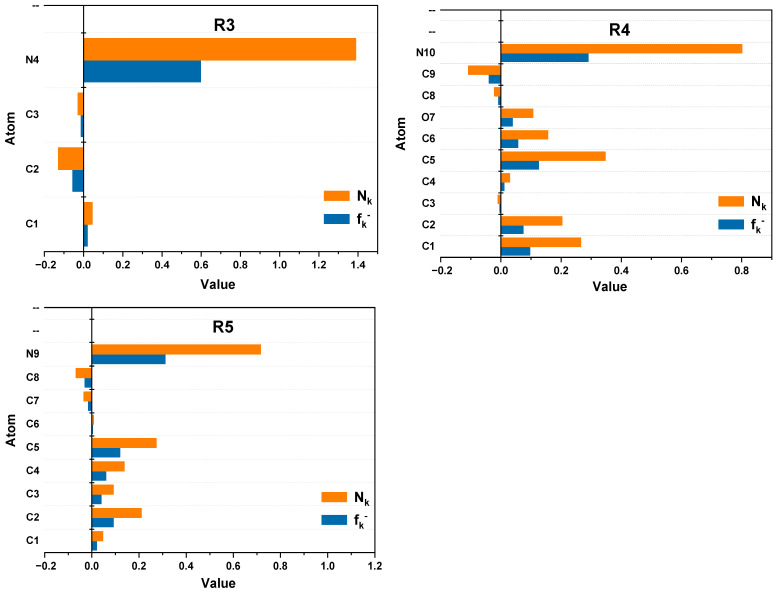
Local Reactivity Analysis—Fukui indices (f_k_^+^ and f_k_^−^), electrophilicity (ω_k_), and nucleophilicity (N_k_) for R1, R2, R3, R4, and R5 based on NPA Population Analysis.

Figure 6 shows a detailed analysis of local reactivity indices, including Fukui indices (f_k_^+^ and f_k_^−^), local electrophilicity (ω_k_), and nucleophilicity (N_k_), for compounds R1, R2, R3, R4, and R5. These descriptors, calculated using DFT/B3LYP 6-31G(d,p) analysis of the NPA population, identify reactive sites susceptible to participation in electrophilic and nucleophilic interactions. For R1, atom C6 exhibits the highest values for both the local electrophilicity index (ω_k_) and the Fukui index (f_k_^+^), indicating its strong predisposition to attract electrons and participate in electrophilic interactions. The nucleophilic properties of the compounds, characterized by N_k_ and f_k_^−^, highlight the main reactive sites of each molecule. R2 exhibits strong nucleophilicity at the N10 atom, with the highest N_k_ and f_k_^−^ values, making it a primary site for electron donation. Similarly, the N4 atom in R3 shows significant N_k_ and f_k_^−^ values, making it highly reactive toward both electrophilic and nucleophilic interactions. R4 follows a similar trend, with the N10 atom displaying the strongest nucleophilic indices. In the case of R5, N9 emerges as the most nucleophilic site, with dominant N_k_ and f_k_^−^ indices, indicating its potential for interactions with strongly electrophilic species. These trends confirm the results obtained previously.

### 2.4. Assessment of the Antimicrobial and Antioxidant Activity of the New Pyrazole Derivatives

#### 2.4.1. Antioxidant Activity

**Figure 7:**
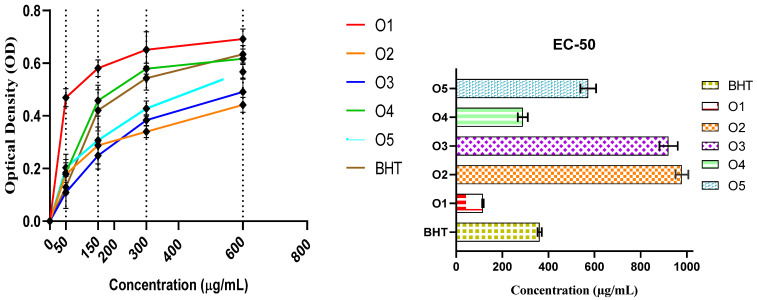
Antioxidant potency of pyrazole derivatives determined using the FRAP method.

Pyrazole compounds are important scaffolds in the development of agents with significant antioxidant activity [31,32]. Therefore, we aimed to evaluate the newly synthesized pyrazole scaffold derivatives for their ferric-reducing antioxidant power by employing the FRAP assay and their free radical scavenging potential using the DPPH test. The results from the FRAP assay (Table 2 and Figure 7), which measures ferric-reducing antioxidant power, show that the reducing ability of the synthetized compounds (O1–5) increases with concentration. At 50 μg/mL, O1 displayed the highest reducing power with an optical density (OD) of 0.4690 ± 0.0343, surpassing even the standard antioxidant BHT, which had an OD of 0.1290 ± 0.0184. As the concentration increased, all compounds showed an improvement in reducing capacity, with O1 continuing to demonstrate the strongest activity, achieving an OD of 0.6920 ± 0.0385 at 600 μg/mL—the highest among all the tested compounds. O 4, although it also displayed significant reducing capacity, reached an OD of 0.6170 ± 0.0172 at 600 μg/mL, which was lower than that of O1 but still substantial. O5 followed closely, with an OD of 0.5670 ± 0.0283 at the same concentration, indicating moderate reducing power. In contrast, O2 and O3 showed weaker antioxidant potential, with O2 reaching an OD of only 0.4420 ± 0.0283 at 600 μg/mL, making it the least effective in this assay. The EC50 values, which represent the concentration at which 50% of the maximum ferric-reducing power is achieved, further highlight the differences in antioxidant potential. O1 exhibited the lowest EC50 value of 115.84 ± 2.25 (µg/mL), confirming its strong reducing capacity, requiring a lower concentration to achieve half of its maximum activity. O4 and O5 also had relatively low EC50 values of 289.11 ± 15.4 (µg/mL) and 572.84 ± 23.78 (µg/mL), respectively, indicating that they possess considerable reducing power. However, O2 and O3 had much higher EC50 values, indicating weaker antioxidant capabilities that require higher concentrations to reach 50% of their maximum reducing potential.

**Figure 8:**
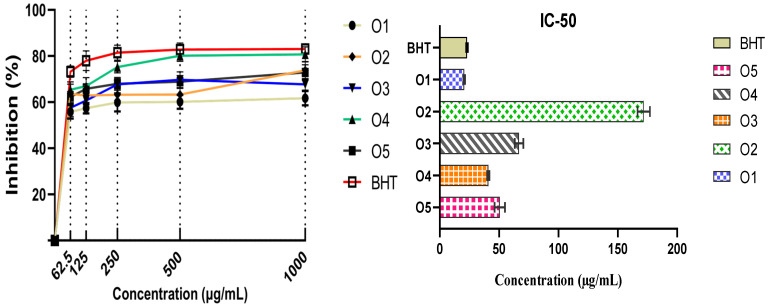
Antioxidant potency of pyrazole derivatives determined by DPPH technique.

The DPPH assay results shown in Table 3 and Figure 8 reveal significant variation in the radical scavenging activity of the O compounds (O1–5), with increasing scavenging efficiency observed as concentration increases. O4 exhibited the highest free radical scavenging activity, achieving 80.72 ± 3.23% inhibition at 1000 μg/mL, which is close to the 83.04 ± 2.20% inhibition of the standard BHT. This makes O4 one of the most effective compounds for DPPH radical scavenging. Similarly, O5 and O3 showed strong inhibition at higher concentrations, with 72.90 ± 3.40% and 67.68 ± 2.54% inhibition, respectively. Despite having the lowest overall free radical scavenging activity at higher concentrations, O1 is noteworthy for its extremely low IC50 value, which signifies its efficacy at lower concentrations. In Table 4, the IC50 values represent the concentrations at which 50% of DPPH radicals were scavenged, and O1 achieved this target at a significantly lower concentration than the other O compounds (20.62 ± 0.46 µg/mL). This suggests that O1 is highly potent in its initial interaction with free radicals, requiring smaller amounts to exhibit a notable antioxidant effect. This efficiency at low concentrations highlights O1 as a fast-acting antioxidant, making it an attractive candidate for applications where rapid and early-stage antioxidant intervention is crucial. In environments or formulations where minimizing oxidative stress at low concentrations is essential, O1 can offer considerable benefits due to its ability to neutralize a significant amount of free radicals without the need for higher concentrations.

While O4 excels at higher concentrations, O1’s potency at lower concentrations should not be overlooked. Its low IC50 value is indicative of its strong antioxidant potential in situations where minimal compound usage is preferred, such as in certain pharmaceutical or cosmetic applications where efficient antioxidant activity at low doses is critical.

#### 2.4.2. Antibacterial and Antifungal Activity

**Figure 9:**
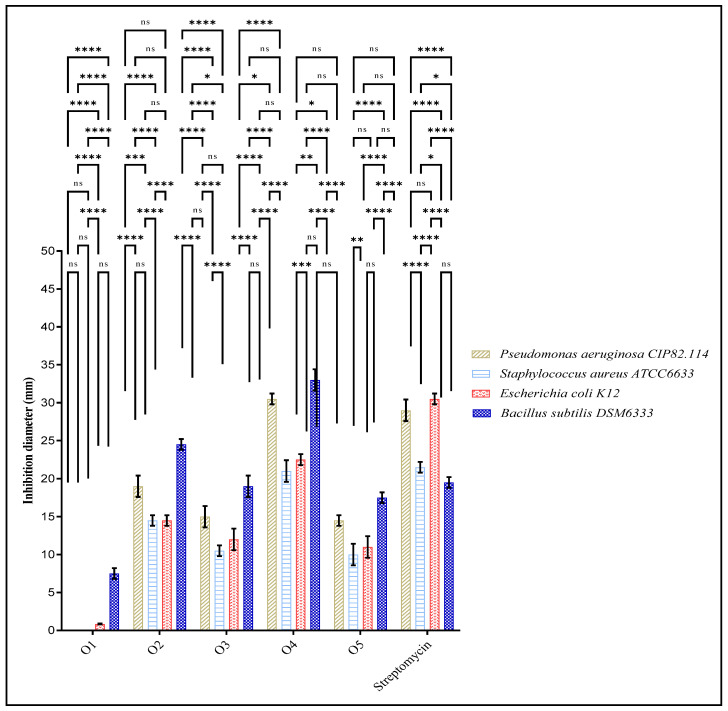
Antimicrobial activity of new pyrazole derivatives against the microbial strains tested. Means (±SDs, *n* = 3) marked with stars indicate significant differences according to two-way ANOVA and Tukey’s multiple-interval tests at *p* < 0.05. ns: “not significant”, *: *p*-value < 0.05, **: *p*-value < 0.01, ***: *p*-value < 0.001, and ****: *p*-value < 0.0001.

Figure 9 highlights the inhibition diameters (in mm) of the pyrazole derivatives (O1 to O5) against various microbial strains, comparing their performance with the reference antibiotic, Streptomycin. O4 showed the highest inhibition diameter (30 ± 0.00 mm) against Pseudomonas aeruginosa, surpassing Streptomycin (29 ± 1 mm), indicating strong antibacterial activity. O2 also demonstrated decent activity (19 ± 1 mm), while O3 and O5 had lower inhibition zones. O1, however, was completely ineffective against Pseudomonas aeruginosa, with an inhibition diameter of 0 mm.

In addition, O4 exhibited identical anti-Staphylococcus aureus ATCC6633 activity to that for Streptomycin, with inhibition diameters of 21 ± 1 mm. O2 followed with a moderate inhibition diameter of 14 ± 0.00 mm, while O3 and O5 exhibited lower activity. Furthermore, for Escherichia coli, O4 showed the best performance with an inhibition diameter of 23 ± 0.00 mm, equal to that for Streptomycin. O2 also showed moderate activity (14 ± 0.5 mm), whereas O3 and O5 displayed weaker inhibition, with values of 12 ± 1 mm and 11 ± 1.00 mm, respectively. O1, once again, showed very minimal activity, with an inhibition zone of only 0.8 mm. Moreover, against Bacillus subtilis, O4 was the most potent, with the highest inhibition zone of 33 ± 1.00 mm, significantly surpassing Streptomycin (19 ± 0.00 mm). O2 also showed strong activity, with an inhibition zone of 24.5 ± 0.5 mm, while O3 and O5 were less effective.

**Figure 10:**
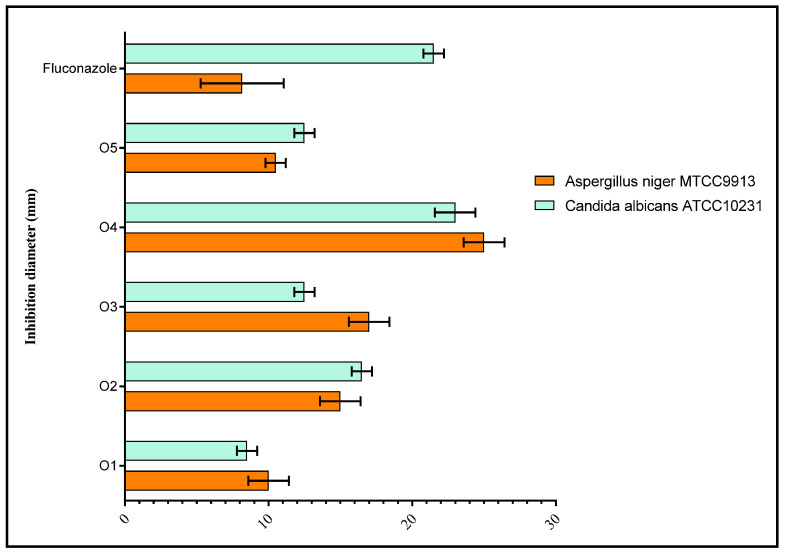
Antifungal activity of pyrazole derivatives against the fungal strains tested.

Likewise, in the case of fungi, the results for which are shown in Figure 10, O4 demonstrated robust anti-*C. albicans* activity with an inhibition diameter of 23 ± 1 mm, surpassing fluconazole’s 21 ± 0.5 mm, while O2 showed a smaller inhibition of 16.5 ± 0.5 mm. Against *A. niger*, O4 displayed even stronger efficacy, with a 25 ± 1 mm inhibition, significantly outperforming fluconazole’s 8.16 ± 2.04 mm. Other O compounds showed inhibition diameters ranging from 10 to 18 mm, further indicating greater antifungal potential than fluconazole across both fungal strains. Overall O2 and O5 showed moderate activity, while O1 consistently displayed the weakest performance across all strains. Consequently, O4 emerged as the most effective compound based on its high inhibition diameters across all microbial and fungal strains.

Based on the MIC and MBC values from Table 5, O4 consistently demonstrated the strongest antimicrobial activity across most strains. For Pseudomonas aeruginosa, O4 had the lowest MIC and MBC values (both 0.129 mg/mL), making it the most potent compound against this strain. In contrast, O1 had the highest MIC and MBC values (0.191 mg/mL), indicating weak activity. Similarly, for Escherichia coli, O4 had the best performance with an MIC of 0.016 ± 0.004 mg/mL, followed closely by O3 with an MIC of 0.033 ± 0.006 mg/mL. O1, again, showed weaker activity against E. coli with an MIC of 0.024 ± 0.006 mg/mL. However, O5 showed the best performance with an MIC of 0.017 ± 0.004 mg/mL against Staphylococcus aureus, but O4 and O3 were nearly as effective, with MIC values of 0.032 ± 0.006 mg/mL and 0.033 ± 0.005 mg/mL, respectively. For Bacillus subtilis, O4 had the lowest MIC (0.016 ± 0.003 mg/mL) and MBC (0.032 ± 0.005 mg/mL), confirming its superior activity. Candida albicans followed a similar trend, with O4 showing the best antifungal activity (MIC and MBC both 0.032 mg/mL), while O3 also performed well, especially for Aspergillus niger, with an MIC and MBC of 0.008 mg/mL. Overall, O4 emerged as the most potent compound, with O3 following closely, while O1 consistently had the weakest activity across the strains.

The current results were similar to those of [33], which reported that several 4-functionalized-pyrazoles were synthesized and showed significant antibacterial activity, especially compound 9e, which exhibited strong antibacterial activity against *Staphylococcus aureus* MTCC96 and *Bacillus subtilis* MTCC121, with MIC values of 64 and 32 μg/mL, respectively, and also to those of [34], which presented a synthesis of several new series of N-aryl-4-(1,3-diaryl-1H-pyrazol-4-yl) thiazol-2-amine derivatives (8a–x). Among the 24 synthesized pyrazolyl–thiazole derivatives, 6 (8a, 8b, 8j, 8n, 8o, and 8s) demonstrated significant activity against *Staphylococcus aureus* NCIM2178. Notably, compounds 8j and 8s displayed potent antibacterial effects, with a minimum inhibitory concentration (MIC) of 0.0625 mg/mL against *S. aureus*. Compound 8s also exhibited moderate activity against *Proteus mirabilis* NCIM 2388, though its exact MIC in mg/mL was not specified, while both compounds showed limited activity against *Escherichia coli* NCIM2574. These results highlight the selective antibacterial potential of compounds 8j and 8s, positioning them as promising candidates for targeted antibacterial applications, especially against *S. aureus*.

In addition, K.B. Gangurde et al. [35] focused on the synthesis of benzotriazole–pyrazole–thiazole hybrid compounds (9a–h), which demonstrated notable antibacterial activity against *Bacillus subtilis* MTCC441, with MIC values ranging from 0.0156 to 0.25 mg/mL. Against *Staphylococcus aureus* MTCC3166, the most active compounds, 9e and 9h, had MIC values of 0.0156 mg/mL, while other compounds showed less potent MICs of up to 0.0625 mg/mL. For *Escherichia coli* (MTCC 40), compound 9g displayed the lowest MIC at 0.0156 mg/mL, whereas 9c exhibited the highest MIC at 0.25 mg/mL. Our results are in contradiction to those of [36], which indicated that various synthesized pyrazole–thiazole derivatives showed important antibacterial activity against *Pseudomonas aeruginosa* MTCC 2642. Compound 6c showed the strongest activity, with a minimum inhibitory concentration (MIC) of 0.005 mg/mL, outperforming the standard ampicillin (MIC of 0.0156 mg/mL).

With regard to antifungal activity, the obtained result is in good agreement with the studies of Nagamallu and colleagues [37]. They synthesized several pyrazole derivatives, and these compounds showed good activity against *Aspergillus niger* and *Candida albicans*, especially Compound 38, which had MICs of 0.0125 mg/mL against *A. niger* and 0.0500 mg/mL against *C. albicans*.

### 2.5. In Silico Physicochemical Features and Drug-likeness Properties

The synthesized compounds exhibit different physicochemical and drug-likeness characteristics that affect their potential for becoming drugs. As shown in Table 6, the compounds differ in molecular weight, volume, and TPSA. O1 has the highest molecular weight and volume, and this may suggest that this compound may more effectively interact with living molecular targets. Meanwhile, O2 has the lowest molecular weight, which means that it may be more absorbable due to its smaller size. The TPSA data indicate that all of the compounds fall below the value of 90 Å^2^, meaning that all the compounds are membrane-permeable. O4 has the highest TPSA value of 48.12 Å^2^, so this compound is also considered to be more water-soluble. However, O3 has the lowest number of rotatable bonds and the lowest lipophilicity, with a LogP value of 2.02, meaning that this compound has an overall better chance in terms of rigidity and an equal solubility-to-permeability ratio. Meanwhile, according to Table 7, all the compounds meet the four essential drug-likeness rules, including the Muegge, Egan, Ghose, and Lipinski rules, which are most relevant in terms of their effectively being absorbed. Only O1 does not meet the Veber rule for being too rigid, as the compound has nine rotatable bonds, meaning that this factor may negatively impact its permeability. Still, O1 meets the other three critical Pfizer, GSK, and Golden Triangle rules, which indicates that the strength of its relatively small molecular size and lipophilicity make the compound less potentially toxic and increase its pharmacokinetic efficacy. Overall, this suggests that the compounds have a good chance in terms of safety and likely success in being developed as drugs. In addition, together, the data prioritize O3 over the rest, considering its acceptable molecular size and lower lipophilicity combined with the optimal number of rotatable bonds. However, considering that O1 is acceptable due to the scales, all the synthesized compounds have strong properties in the context of prospective drug development.

### 2.6. Prediction of Computational ADMET

To be a good drug candidate, not only does a molecule need to be potent at low doses, with minimum toxicity, it also needs to have appropriate pharmacokinetic properties to ensure that the drug is present in the relevant organism for a sufficient time to operate and avoid accumulation [38]. Thus, it is vital to investigate the ADMET aspects of a new drug. Employing the first in silico technique to examine ADMET properties of a drug candidate allows for effective drug-seeking drug production before in vivo or in vitro education takes place. ADMET properties are also important in terms of a drug’s design. Indeed, in silico forecasting of ADMET may substantially decrease the likelihood of premature outcomes in the final phase of marketing.

The compound absorption profile results for the O compounds imply a strong uptake in the intestine, with each of the values higher than 92%, as shown in Table 8. O1 has the highest of the values, 97.46%, followed by O4, which shows a 97.389% absorption rate. Such high absorption means that every compound could be effectively administered orally [39], as they are likely to be efficiently absorbed through the human intestine. For the volume of the distribution, VDss, the results vary by compound, but O1 and O2 appear to have higher values, 1.294 and 1.156 log L/kg, respectively. VDss measures the disposition of a compound and is an indicator of its distribution in the tissues of the body. Higher values may be beneficial for drugs with targets in tissues, but they could also mean an increased retention in the tissue, eliminating the possibility of metabolic elimination [40]. As for the BBB permeability, O2, O3, and O5 have slightly positive values, with a log BB from 0.109 to 0.192, which may suggest that the compounds can cross the blood–brain barrier to some extent. According to the central nervous system (CNS) score, compounds with a LogPS > −2 are considered capable of penetrating the CNS, while compounds with a LogPS < −3 are considered incapable of penetrating the CNS [41]. The distribution indices reported for the new pyrazole derivatives show that O4 and O5 can penetrate the CNS; however, the rest of the compounds have less potential to penetrate it. The metabolic characteristics of the O compounds involve interactions with essential cytochrome P450 enzymes, which are highly responsible for drug metabolism [42]. Notably, none of the O compounds can serve as a substrate for CYP2D6, thereby reducing the level of interaction with other drugs that are metabolized by the said enzyme. However, O1, O4, and O5 are substrates for CYP3A4, which implies that the compounds might be metabolized by the enzyme, a common process in recent drug processing. Moreover, O1, O3, and O5 inhibit CYP2D6, which might translate to potential drug interactions if the compounds are administered with other drugs that are metabolized by CYPD26. O4 inhibits CYPC9 as well as CYP1A2, alongside both O4 and O5, suggesting that they might interact with drugs that are metabolized by either of the two enzymes. Notably, none of the drugs inhibit CYP3A4, which implies that they cannot interact with other drugs that are metabolized by the same path. In the excretion category, the O compounds have moderate clearance rates. O2’s clearance is the highest, suggesting that it will be eliminated first from the body. O3 and O4 will be expected to be retained for longer, thus delivering the drug; however, there will be higher risks of accumulation if used for too long. Finally, with regard to toxicity, none of the O compounds display AMES toxicity, indicating that they are unlikely to be mutagenic. This is particularly important for assuring the safety of the compounds, as there is a very low probability of long-term carcinogenic effects. The favorable toxicity profile for all the compounds makes them prime candidates for further drug development.

### 2.7. Analysis of Molecular Docking

The docking study explored antioxidant, antibacterial, and antifungal potentials by targeting the proteins 2CAG, 3FV5, and 1EA1, respectively, highlighting how O compounds bind to critical residues to stabilize these reactions. The 2CAG protein from *Proteus mirabilis* is a catalase enzyme crucial for reducing cellular oxidative stress by breaking down hydrogen peroxide into water and oxygen [43]. This function is facilitated by its iron-bound heme group, which undergoes a two-step oxidation reaction to neutralize harmful reactive oxygen species, thereby supporting cellular antioxidant defenses. The 3FV5 protein, on the other hand, is a structural model of *E. coli* Topoisomerase IV, an enzyme involved in DNA replication by managing DNA topology through strand breaks and rejoining [44]. By regulating DNA supercoiling, Topoisomerase IV is essential for bacterial cell division, making it an ideal target for antibacterial drug development. Inhibiting this protein disrupts bacterial DNA replication, which can significantly reduce bacterial viability and pathogenicity [45]. The 1EA1 protein is crucial in molecular docking studies for designing and evaluating new inhibitors targeting CYP51, an essential enzyme in *Mycobacterium tuberculosis* and a prime target for developing antifungal and antitubercular agents [46]. Its crystal structure, resolved in complex with fluconazole, provides detailed insights into how inhibitors bind to CYP51, guiding the optimization of molecular scaffolds to enhance potency and specificity in drug development.

**Figure 11:**
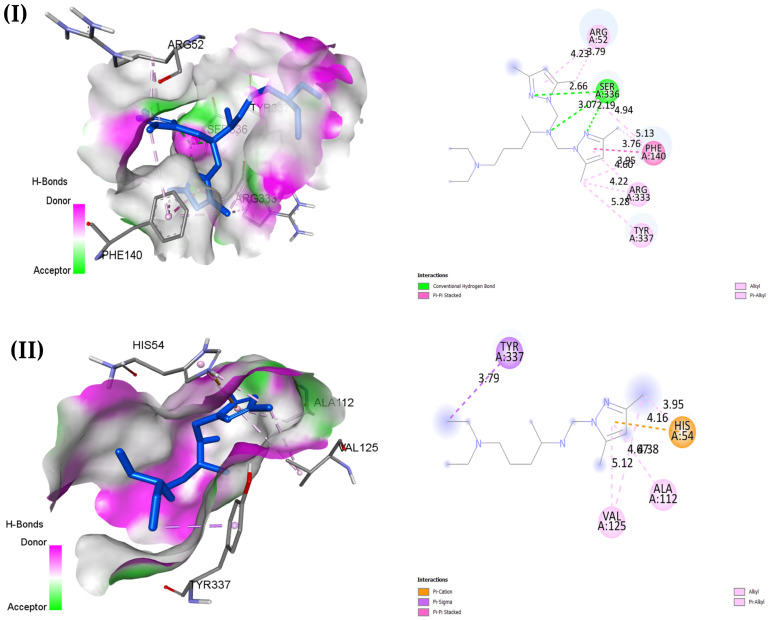

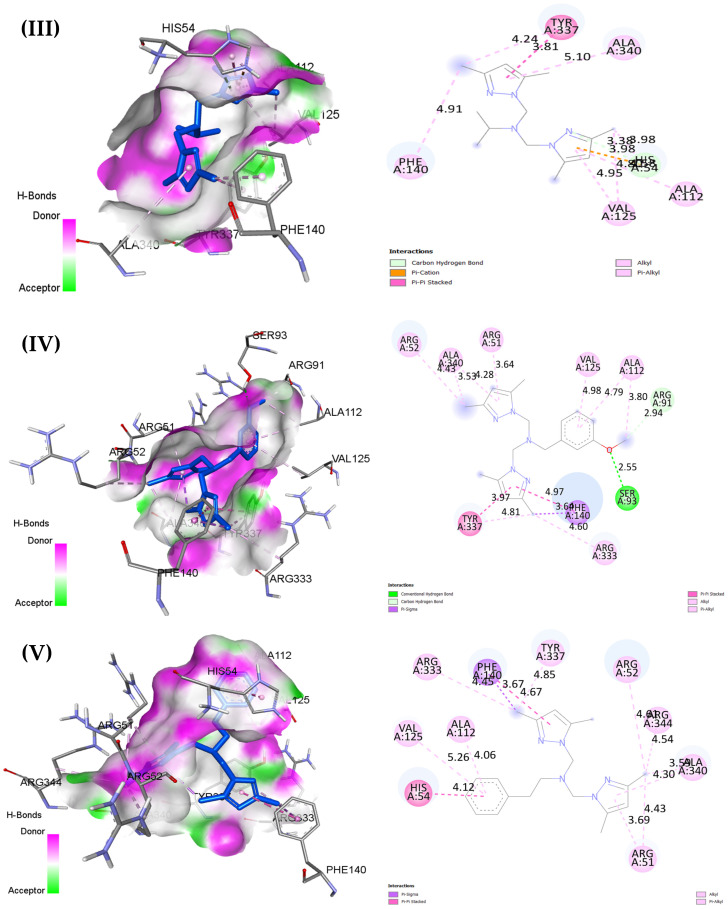

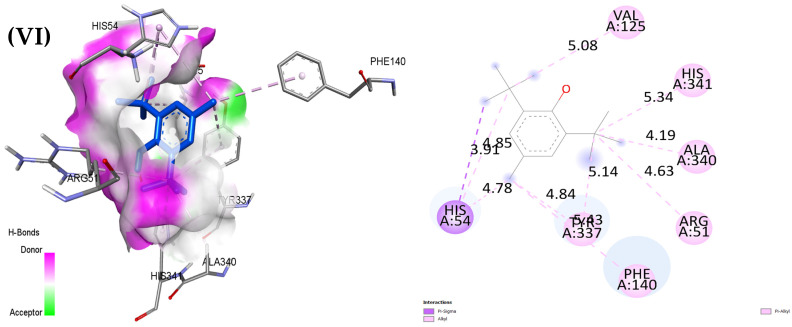
Two- and three-dimensional docked views of O1–O5 (**I**–**V**) and BHT (**VI**) with active site of 2CAG protein.

In the antioxidant activity with the 2CAG protein shown in Table 9 and Figure 11, O5 has the strongest binding energy of −8.7 kcal/mol, forming hydrophobic π–alkyl and alkyl interactions with residues like ARG333 and ALA112, while PHE140 forms π–sigma interactions with the methyl group of the pyrazole ring. Although it does not form hydrogen bonds, O5’s strong binding energy outperforms BHT, with a binding energy of −7.3 kcal/mol, which suggests it has high potential as an antioxidant agent. O1, with a slightly lower binding energy of −7.6 kcal/mol, compensates by forming three hydrogen bonds with SER336 at distances of around 2.19–3.07 Å, adding stability that BHT lacks, which could enhance binding stability. O3 and O4 also exhibit strong binding affinities at −8.1 and −8.4 kcal/mol, respectively, engaging in stabilizing interactions without hydrogen bonds. Overall, O1’s hydrogen bonding network makes it a strong antioxidant candidate, while O5, with the highest binding energy, also shows substantial promise.

**Figure 12:**
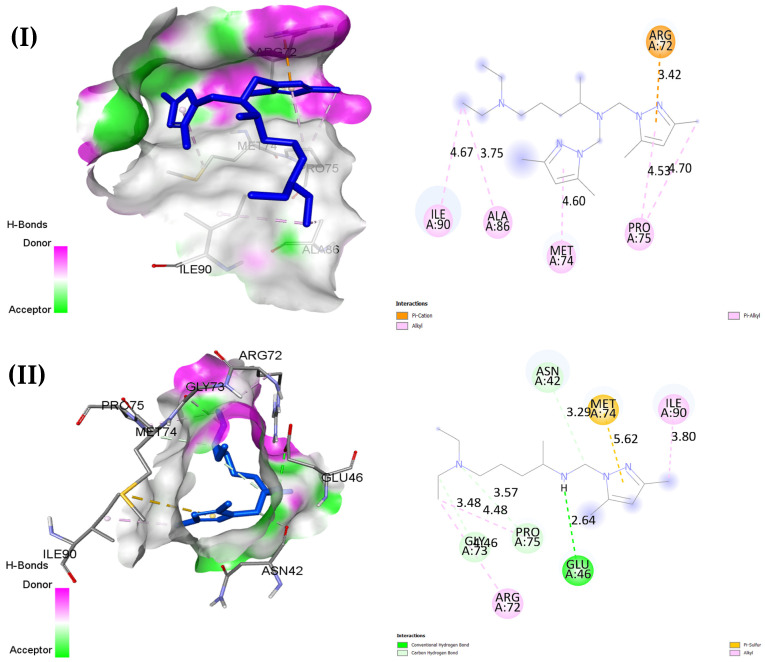

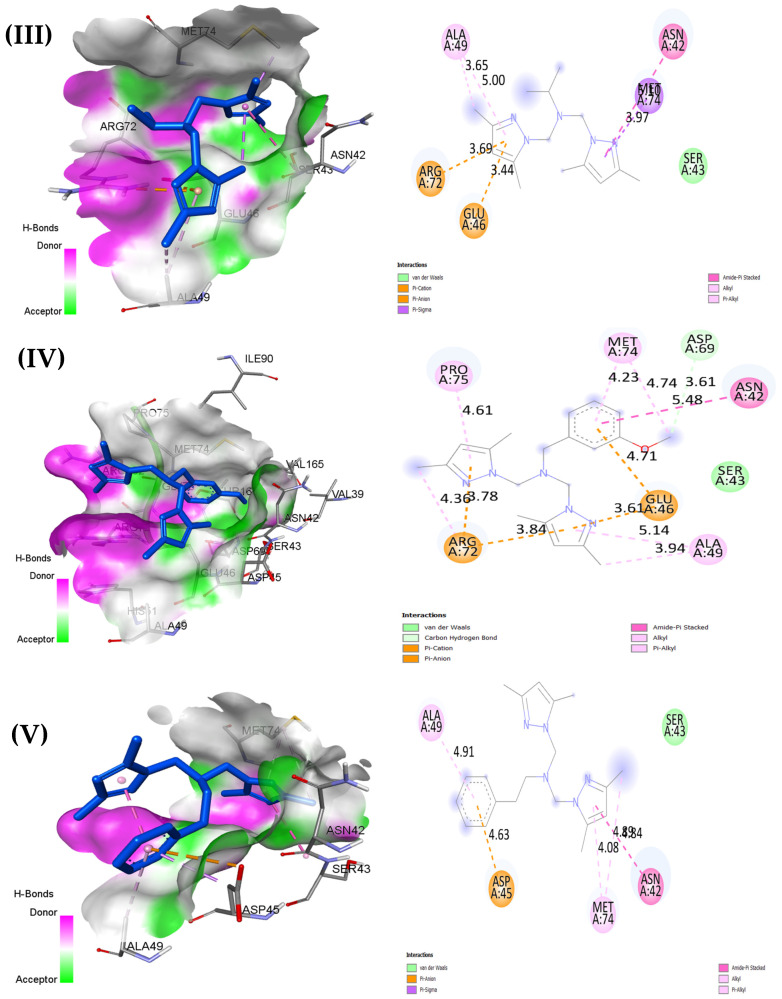

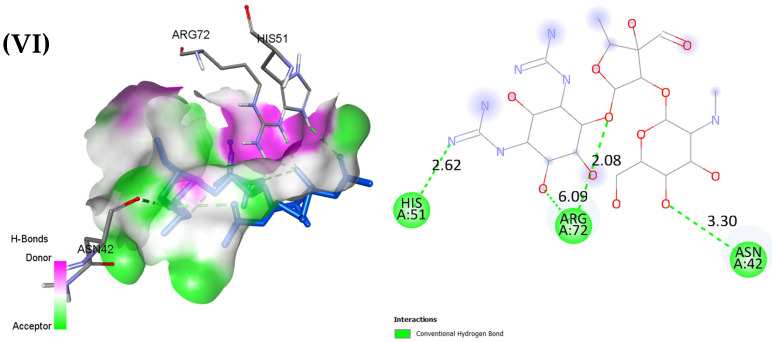
Two- and three-dimensional docked views of O1–O5 (**I**–**V**) and Streptomicyne (**VI**) with active site of 3FV5 protein.

In the antibacterial docking analysis with 3FV5 represented in Table 10 and Figure 12, O4 shows the highest binding affinity at −6.0 kcal/mol, forming electrostatic interactions with GLU46 and ARG72 and exhibiting carbon–hydrogen bond interplay with ASP69 and interacting with many hydrophobic types with the rest of the residues; also, it has a stronger binding affinity compared to streptomycin’s −5.4 kcal/mol. Despite not forming hydrogen bonds, while streptomycin stabilizes via such bonds, these interactions make O4 the leading antibacterial candidate due to its stable binding profile. O3 follows closely, with a binding energy of −5.3 kcal/mol, engaging residues such as ARG72 and GLU46, but also lacks hydrogen bonds. O5 and O1 exhibit binding energies of −5.3 kcal/mol and −5.0 kcal/mol, respectively, making electrostatic and hydrophobic contacts with key residues like ALA49 and MET74. O2 has the lowest binding affinity at −4.3 kcal/mol but does form a stabilizing conventional hydrogen bond with GLU46 and a carbon–hydrogen bond with ASN42. These findings suggest that O4 and O3 are the most promising antibacterial agents, with O2 offering additional stability through hydrogen bonding.

**Figure 13:**
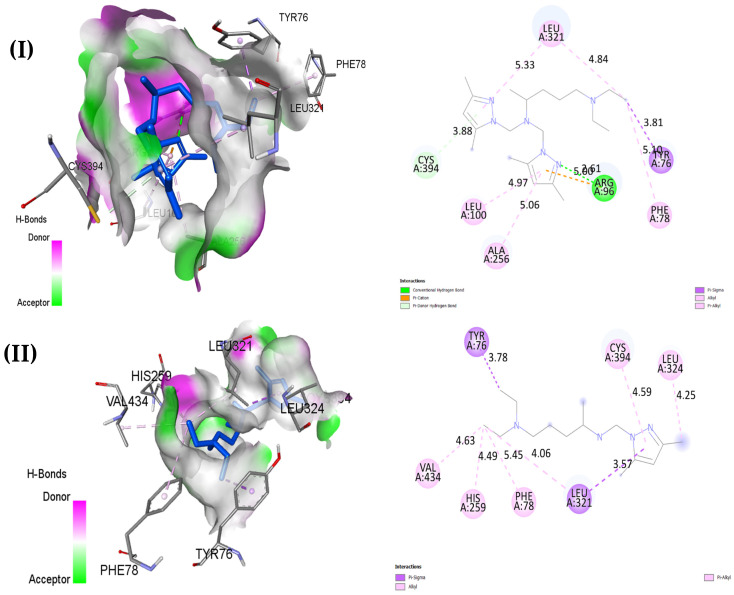

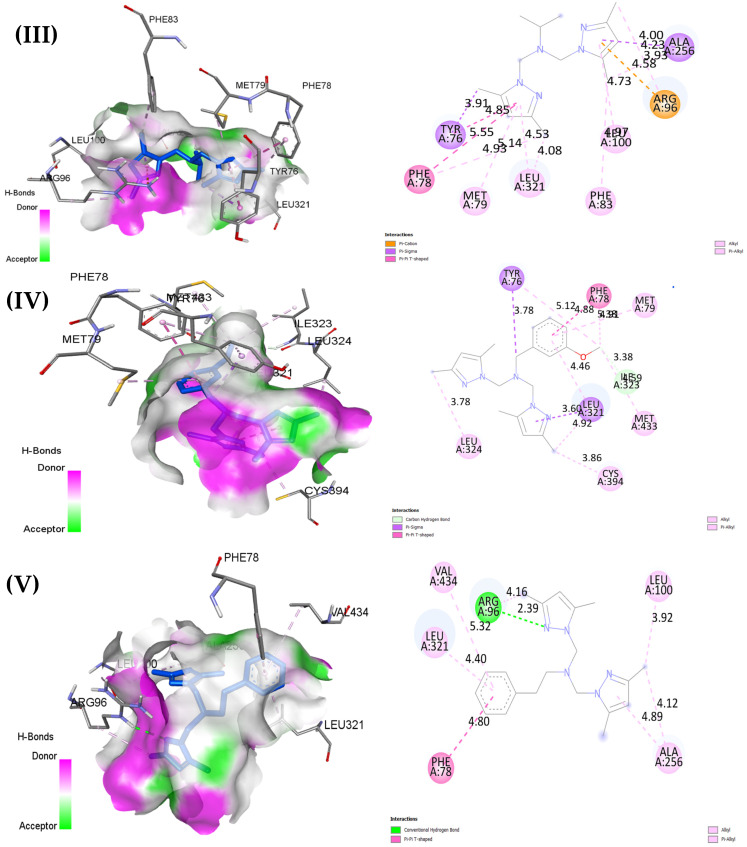

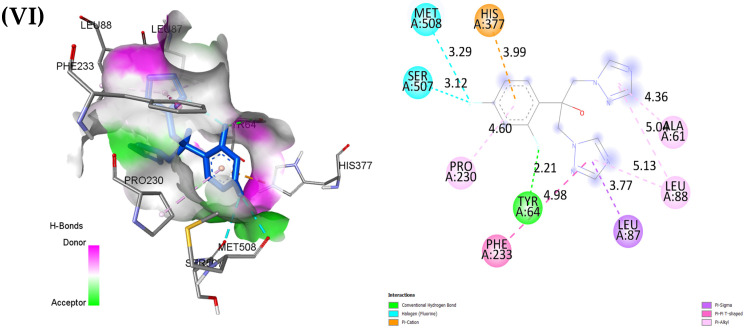
Two- and three-dimensional docked views of O1–O5 (**I**–**V**) and Fluconazole (**VI**) with active site of 1EA1 protein.

**Figure 14:**
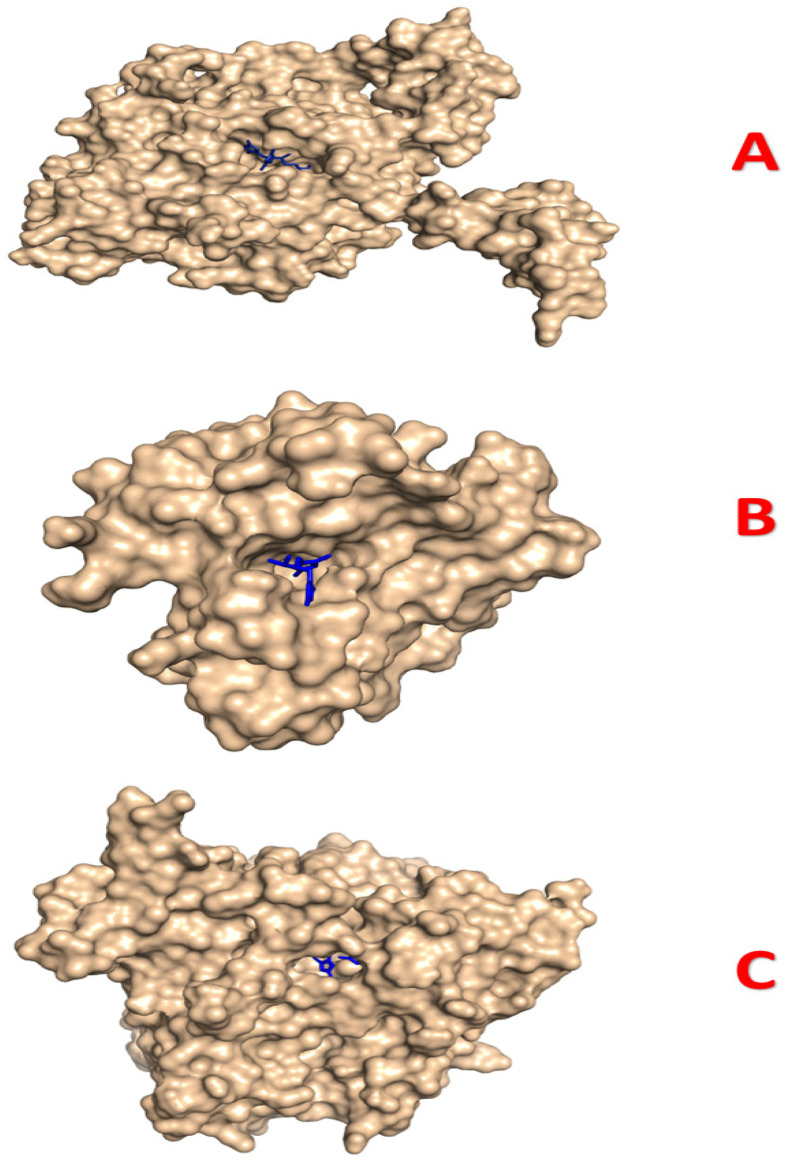
Three-dimensional visualization of the complex O 1—2CAG (**A**), the complex O 4—3FV5 (**B**), and the complex O 4—1EA1 (**C**) created using Pymol. color blue: Ligand.

Regarding the antifungal docking analysis with 1EA1 reflected in Table 11 and Figure 13, O4 exhibits the highest binding affinity at −8.5 kcal/mol, forming hydrophobic interactions, such as π-σ, π–alkyl, and π-π T-shaped interactions, with residues like TYR76, LEU324, CYS394, and PHE78, as well as a carbon–hydrogen bond interaction with ILE323 at a distance of 4.59 Å, while O5 follows closely behind in binding energy (−8.4 Kcal/mol), establishing similar types of predominant hydrophobic interactions to those described above, alongside a conventional hydrogen bond interaction with ARG96. Fluconazole (−7.0 kcal/mol), also forming a hydrogen bond, was found to possess a higher specificity score, with an additional two halogen bonds with fluorine interacting with residues SER507 and MET508, ensuring its stability upon binding (Figure 14. Three-dimensional visualization of the complex O 1—2CAG (**A**), the complex O 4—3FV5 (**B**), and the complex O 4—1EA1 (**C**) created using Pymol). On the other hand, O1, O2, and O3 show weaker binding affinities (−6.4 kcal/mol, −5.6 kcal/mol, and −6.7 kcal/mol of free energy, respectively); only one hydrogen bond was formed by O1, and the others were sustained by only hydrophobic forces. These three ligands frequently interact with residues like LEU321, PHE78, and TYR76, but the absence of hydrogen bonds in O2 and O3 may explain their relatively lower binding affinities, underscoring the importance of hydrogen bonding in stabilizing ligand–protein interactions.

## 3. Materials and Methods

### 3.1. Items and Materials

Merck reagents were employed with no further purification. Analysis included ^13^C NMR and ^1^H NMR experiments carried out on a Bruker AM 300 spectrometer, operating at 75.47 MHz for 13C and 300.13 MHz for 1H. CDCl_3_ and DMSO-d6 were used as solvents, and NMR data were reported in parts per million (δ), while coupling constants were referenced to TMS and expressed in Hz. The mass spectrometry was carried out on the Exactive orbitrap mass spectrometer Thermo Fisher (The equipment was sourced from Thermo Fisher Scientific, Waltham, MA, USA) using the electron ionization method (direct sample introduction). Melting points were determined with the help of the Koffler bank.

### 3.2. Synthesis of Pyrazole Drug Candidate Derivatives

#### 3.2.1. N4,N4-bis((3,5-Dimethyl-1H-pyrazol-1-yl)methyl)-N1,N1-diethylpentane-1,4-diamine (O1)

**Figure 15:**
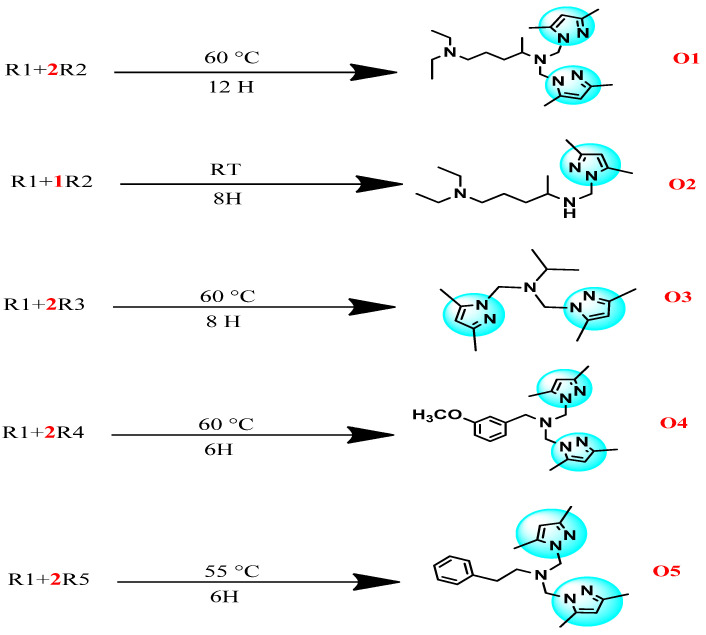
Synthesis method of novel pyrazole derivatives O1–O5.

As described in Figure 15, N1,N1-diethylpentane-1,4-diamine (R2) (3 mL, 15.19 mmol) and (3,5-dimethyl-1H-pyrazol-1-yl)methanol (R1) (3.89 g, 30.38 mmol) were stirred jointly into acetonitrile (20 mL) under 60 C for 12 h, followed by evaporation of the solvent, then washing with diethyl ether, and the same steps were repeated to obtain the final product (oil: 5.44 g; yield: 76%); 1H NMR (300.13 MHz, CDCl_3_): δ (ppm) = 0.66(2CH3, 6H, dd); 0.95(CH3, 3H, m); 1.87(2CH3, 6H, q); 1.94(2CH3, 6H, s); 2.10(2H, m); 2.13(2CH2, 4H, m); 2.60(4H, q); 4.58(4H, m); 5.1(1H, s); 5.45(2H, d); 13C-NMR (75.47 MHz, CDCl_3_): δ (ppm) = 10.1(2CH3); 11.8(2CH3); 13.9(2CH3); 16.1(CH3); 24.2(CH2); 32.1(CH2); 47.2(2CH2); 52.5(2CH2); 62.1(2CH2); 105.2(2C_pyr_=C); 138.9(2C_pyr_=C); 147.3(2C=N). Boiling point: 103–104 °C MS (EI): M+. = 374.31583.

The compound N4,N4-bis((3,5-dimethyl-1H-pyrazol-1-yl)methyl)-N1,N1-diethylpentane-1,4-diamine (O1) was synthesized with a yield of 76%. The 1H NMR spectrum shows several distinct signals: the doublet of doublets at δ 0.66 (2CH3, 6H) corresponds to methyl groups attached to a carbon near other substituents. The signal at δ 0.95 (CH3, 3H, m) is attributed to the terminal methyl of an ethyl group, while the quartet at δ 1.87 (2CH3, 6H) and the singlet at δ 1.94 (2CH3, 6H) indicate methyl groups within the pyrazolylmethyl substituents. The multiplet at δ 2.10 (2H, m) and δ 2.13 (2CH2, 4H, m) confirms methylene protons in the alkyl chain, which is part of the backbone connecting the pyrazole group to the pentane chain. Further signals, such as δ 4.58 (4H, m) and δ 5.10 (1H, s), correspond to methylene and pyrazole protons, confirming the presence of the pyrazole ring and its substitution pattern. The 13C NMR spectrum shows the expected signals for methyl and methylene groups, with peaks at δ 10.1, δ 11.8, and δ 13.9 indicating methyl carbons and those at δ 105.2, δ 138.9, and δ 147.3 confirming the pyrazole ring’s aromatic and functional carbons. The molecular ion peak at *m*/*z* 374.31 in the MS spectrum corroborates the molecular weight, confirming the identity of the compound. Overall, the NMR and MS data are consistent with the proposed structure of O1, containing two pyrazole rings attached to the alkyl chain (Figures S1–S3).

#### 3.2.2. N4-((3,5-Dimethyl-1H-pyrazol-1-yl)methyl)-N1,N1-diethylpentane-1,4-diamine (O2)

N1,N1-diethylpentane-1,4-diamine (R2) (3 mL, 15.19 mmol) and (3,5-dimethyl-1H-pyrazol-1-yl)methanol (R1) (1.94 g, 15.19 mmol) were added dropwise then stirred jointly into acetonitrile (20 mL) at room temperature for 8 h, followed by evaporation of the solvent and washing with diethyl ether, and the same steps were repeated to obtain the final product (oil: 2.87g; yield: 71%); 1H NMR (300.13 MHz, CDCl_3_): δ (ppm) = 0.90(2CH3, 6H, t); 1.17(3H, m); 1.30(4H, m); 2.00(3H, d); 2.15(5H, CH3-CH2, m); 2.38(4H, m, 2CH2); 2.63(CH, 1H, m); 4.81(N-CH2-, 2H, m); 5.38(NH, 1H, s); 5.69(1H_pyr_, s); 13C-NMR (75.47 MHz, CDCl_3_): δ (ppm) = 10.2(CH3); 11.8(2CH3); 12.5(CH3); 21.1(CH3); 23.6(CH2); 32.1(CH2); 47.9(2CH2); 52.6(CH); 53.4(CH2); 62.4(CH2-NH); 105.8(C_pyr_=C); 139.6(CH3-C_pyr_=C); 149.1(CH3-C_pyr_=N). Boiling point: 90–91 °C MS (EI): M+. = 266.24701.

The compound O2 was synthesized with a yield of 71%. The 1H NMR spectrum reveals several key features: the doublet of triplets at δ 0.90 (2CH3, 6H, t) corresponds to the two methyl groups from the ethyl substituents. The multiplet at δ 1.17 (3H, m) is assigned to the methylene protons of the ethyl group, while that at δ 1.30 (4H, m) corresponds to additional methylene groups in the chain. The singlet at δ 2.00 (3H, d) suggests a methyl group attached to a nitrogen atom, and that at δ 2.15 (5H, CH3-CH2, m) is indicative of the ethyl group attached to the nitrogen. The δ 2.38 (4H, m, 2CH2) signal indicates methylene groups adjacent to nitrogen, and that at δ 2.63 (CH, 1H, m) corresponds to a single proton, likely on a pyrazole group. The singlet at δ 5.38 (NH, 1H, s) represents the amine hydrogen, while that at δ 5.69 (1Hpyr, s) corresponds to a pyrazole proton. In the 13C NMR, methyl groups are seen at δ 10.2, δ 11.8, and δ 12.5, confirming the presence of several methyl substituents. The aromatic carbons of the pyrazole ring are visible at δ 105.8 (Cpyr=C) and δ 139.6 (CH3-Cpyr=C), confirming the pyrazole structure. The molecular ion peak at *m*/*z* 266.24701 supports the molecular weight, confirming the identity of O2. Overall, the NMR data indicate a structure containing both ethyl groups and pyrazole rings, with the MS confirming the molecular weight (Figures S4–S6).

#### 3.2.3. N,N-bis((3,5-Dimethyl-1H-pyrazol-1-yl)methyl)propan-2-amine (O3)

Propan-2-amine (R3) (7 g, 11.9 mmol) and (3,5-dimethyl-1H-pyrazol-1-yl)methanol (R1) (3 g, 23.8 mmol) were stirred jointly into acetonitrile (20 mL) under 60 C for 8 h, followed by evaporation of the solvent and washing with diethyl ether, and the same steps were repeated to obtain the final product (white solid: 2 g; yield: 61%); 1H-NMR (300.13 MHz, CDCl_3_): δ (ppm) = 1.04(2CH3, 6H, d); 2.08(2CH3, 6H, s); 2.20(2CH3, 6H, s); 3.16(CH, 1H, m); 4.88(2CH2, 4H, s); 5.79(2(-CH=C), 2H, s); 13C-NMR (75.47 MHz, CDCl_3_): δ (ppm) = 10.6(2CH3); 13.7(2CH3); 19.8(2CH3); 54.6(CH); 62.2(2CH2); 105.1(2C_pyr_=C); 139.9(2N-C_pyr_=C); 147.8(2C_pyr_=N). mp: 86–87 °C MS (EI): M+. = 275.21137.

The synthesis of O3 resulted in a yield of 61%. The 1H NMR spectrum shows signals at δ 1.04 (2CH3, 6H, d) for the methyl groups attached to the nitrogen and at δ 2.08 (2CH3, 6H, s) and δ 2.20 (2CH3, 6H, s) for additional methyl groups in the pyrazole rings. The multiplet at δ 3.16 (CH, 1H, m) is assigned to a proton in the alkyl chain. The singlet at δ 4.88 (2CH2, 4H, s) represents methylene groups attached to nitrogen, and the signal at δ 5.79 (2(-CH=C), 2H, s) corresponds to pyrazole protons. In the 13C NMR, the signals at δ 10.6, δ 13.7, and δ 19.8 correspond to methyl groups, while those at δ 54.6 (CH) and δ 62.2 (2CH2) confirm the presence of the alkyl chain and methylene groups. The aromatic carbons are confirmed by signals at δ 105.1 (2Cpyr=C) and δ 139.9 (2N-Cpyr=C). The molecular ion peak at *m*/*z* 275.21137 is consistent with the calculated molecular weight of the compound. The structure consists of two pyrazolylmethyl groups attached to a propan-2-amine backbone, as confirmed by the NMR and MS data (Figures S7–S9).

#### 3.2.4. 1-(3,5-Dimethyl-1H-pyrazol-1-yl)-N-((3,5-dimethyl-1H-pyrazol-1-yl)methyl)-N-(3-methoxybenzyl)methanamine (O4)

3-Methoxybenzylamine (R4) (1.63 g, 11.9 mmol) and (3,5-dimethyl-1H-pyrazol-1-yl)methanol (R1) (3 g, 23.8 mmol) were mixed together in acetonitrile (20 mL) under 60 C for 6 h, followed by evaporation of the solvent and washing with diethyl ether, and the same steps were repeated to obtain the final product (oil: 3.15 g; yield: 75%); 1H-NMR (300.13 MHz, CDCl_3_): δ (ppm) = 2.04(2CH3, 6H, s); 2.23(2CH3, 6H, s); 3.66(CH2-N, 2H, s); 3.77(CH3O, 3H, s); 4.95(2(N-CH2-N), 4H, s); 5.80(2CHpyr, 2H, s); 6.75–7.18(4Harom, 3m); 13C-NMR (75.47 MHz, CDCl_3_): δ (ppm) = 10.6(2CH3); 13.7(2CH3); 52.1(O-CH3); 55.2(CH2); 65.1(2N-CH2-N); 105.2(2Cpyr=C); 113.2(CHarom); 121.3(CHarom=C); 129.8(CHarom); 140.1(2N-Cpyr=C); 140.9(CHarom); 147.8(2Cpyr=N); 160.0(Carom-O). Boiling point: 109–110 °C MS (EI): M+. = 353.22157.

For O4, synthesized with a 75% yield, the 1H NMR shows a singlet at δ 2.04 (2CH3, 6H, s) and δ 2.23 (2CH3, 6H, s) corresponding to methyl groups in the pyrazole rings. The singlet at δ 3.66 (CH2-N, 2H, s) corresponds to methylene protons attached to nitrogen, while that at δ 3.77 (CH3O, 3H, s) represents the methoxy group. The singlet at δ 4.95 (2(N-CH2-N), 4H, s) corresponds to the methylene protons adjacent to nitrogen. The pyrazole protons are seen at δ 5.80 (2CHpyr, 2H, s), and the aromatic protons from the 3-methoxybenzyl group are visible between δ 6.75 and 7.18 (4Harom, 3m). In the 13C NMR, signals at δ 10.6, δ 13.7, and δ 52.1 (O-CH3) confirm the methyl and methoxy groups. The pyrazole ring carbons are observed at δ 105.2 (2Cpyr=C) and δ 140.1 (2N-Cpyr=C). The MS data (*m*/*z* 353.22157) confirm the molecular weight. The NMR and MS spectra indicate a molecule consisting of a pyrazole ring, a methoxybenzyl group, and a methanamine backbone (Figures S10–S12).

#### 3.2.5. N,N-bis((3,5-Dimethyl-1H-pyrazol-1-yl)methyl)-2-phenylethanamine (O5)

2-phenylethanamine (R5) (1.44 g, 11.9 mmol) and (3,5-dimethyl-1H-pyrazol-1-yl)methanol (R1) (3 g, 23.8 mmol) were stirred jointly into acetonitrile (20 mL) under 55 C for 6 h, and the solvent was evaporated, washed with diethyl ether, and then the solvent was added again and evaporated to obtain the final product (oil: 3.1 g; yield: 77%). 1H-NMR (300.13 MHz, CDCl_3_): δ (ppm) = 2.12(2CH3, 6H, s); 2.23(2CH3, 6H, s); 2.58(CH2-Ar, 2H, t); 2.92(CH2-N, 2H, t); 4.93(2CH2 (N-CH2-N), 4H, s); 5.81(2CH_pyr_, 2H, s); 7.02–7.20(5H_arom_, 3m); 13C-NMR (75.47 MHz, CDCl_3_): δ (ppm) = 10.6(2CH3); 13.7(2CH3); 33.1(CH2); 51.8(CH2); 66.8(2N-CH2-N); 105.2(2Cpyr=C); 125.8(CH_arom_=C); 128.6(2CH_arom_=C); 129.1(2CH_arom_=C); 139.9(C_arom_); 140.1(2N-C_pyr_=C); 147.7(2C_pyr_=N). Boiling point: 107–108 °C MS (EI): M+. = 337.22651.

The O5 compound was synthesized with a yield of 77%. The 1H NMR spectrum shows the singlets at δ 2.12 (2CH3, 6H, s) and δ 2.23 (2CH3, 6H, s) corresponding to the methyl groups in the pyrazole rings. The multiplet at δ 2.58 (CH2-Ar, 2H, t) corresponds to methylene protons adjacent to the aromatic ring, while that at δ 2.92 (CH2-N, 2H, t) represents methylene groups adjacent to nitrogen. The singlet at δ 4.93 (2CH2 (N-CH2-N), 4H, s) corresponds to the methylene groups next to nitrogen. The pyrazole protons are seen at δ 5.81 (2CHpyr, 2H, s), and the aromatic protons are found between δ 7.02 and 7.20 (5Harom, 3m). In the 13C NMR, the methyl groups are observed at δ 10.6, δ 13.7, and δ 33.1 (CH2). The signals at δ 105.2 (2Cpyr=C) and δ 125.8 (CHarom=C) confirm the presence of the pyrazole and phenyl groups. The MS data (*m*/*z* 337.22651) confirm the molecular weight of O5. The structure consists of two pyrazolylmethyl groups attached to a 2-phenylethanamine backbone, confirmed by the NMR and MS spectra (Figures S13–S15).

### 3.3. DFT Methodology 

In this study, global reactivity descriptors were calculated using Density Functional Theory (DFT) to assess the electronic and reactivity properties of the compounds. These descriptors, derived from the energies of the Highest Occupied Molecular Orbital (HOMO) and Lowest Unoccupied Molecular Orbital (LUMO), were obtained using the B3LYP/6-31G(d, p) basis set without any geometrical constraints in the gas phase. The calculated values helped to confirm the proposed synthesis routes of the studied compounds. Chemical potential (µ) represents the tendency of a molecule to exchange electrons with the environment. A lower chemical potential indicates higher stability in terms of electron transfer. Hardness (η), defined as the difference between the HOMO and LUMO energies, measures the resistance of a molecule to electron cloud distortion. Higher hardness implies a greater resistance to electronic deformation, indicating a more stable structure. The electrophilicity index (ω) indicates the ability of a molecule to accept electrons. Higher electrophilicity suggests a compound’s tendency to act as an electron acceptor in chemical reactions. The nucleophilicity index (N), defined relative to tetracyanoethylene (TCE) as a reference, is calculated using the HOMO energy of each molecule. This parameter identifies the electron-donating ability of compounds, with higher values indicating stronger nucleophilic behavior. All those parameters are presented in the following Equations [47,48]:

$$\mu =\frac{\left(E_{HOMO}+E_{LUMO}\right)}{2}$$

$$\eta =E_{HOMO}-E_{LUMO}$$

$$\omega =\frac{\mu^{2}}{2\eta }$$

$$N=E_{HOMO}(nucleophile)-E_{HOMO}(TCE)$$

Fukui functions for a molecule can be calculated by the following Equations [49]:

$$f_{k}^{+}=P_{k}(N+1)-P_{k}(N)$$

$$f_{k}^{-}=P_{k}(N)-P_{k}(N-1)$$

where P_k_ (N), P_k_ (N − 1), and P_k_ (N + 1) represent the electronic populations of atom k in the neutral, cationic, and anionic forms of the molecule, respectively.

The local electrophilic (ω_k_) and nucleophilic (N_k_) indices at a specific site k in a molecule are calculated using the following expressions [50]:

$$\omega_{k}=\omega \times {f_{k}}^{+}$$

$$N_{k}=N\times {f_{k}}^{-}$$

### 3.4. Biological Activities

#### 3.4.1. Inspection of Antioxidant Activity

##### Test of Free Radical Scavenging

The antioxidant efficacy of the substances tested was assessed using the DPPH radical scavenging assay. A fresh methanolic solution of 1,1-diphenyl-2-picrylhydrazyl (DPPH) with a concentration of 0.004% was produced and kept at room temperature in the dark to prevent deterioration. Different concentrations of the synthesized compounds (62.5, 125, 250, 500, and 1000 μg/mL) were formulated in conjunction with butylated hydroxytoluene (BHT) as a positive control. A 100 µL batch of each concentration was combined with 750 µL of DPPH solution and incubated for 30 min in a dark environment. Then, the absorbance was quantified at 517 nm using a UV–visible spectrophotometer [51]. The DPPH solution devoid of test samples served as a negative control, while 95% methanol was used as blank. Antioxidant activity was quantified as percentage DPPH radical scavenging, determined using the following equation:

(1) $$\%Inhibition=\left[\frac{\left(A_{f}-A_{i}\right)}{A_{f}}\right]\times 100$$

where A_f_ stands for the absorbance of the reaction control and A_i_ for the absorbance in the presence of the tested chemical. By graphing the relationship between the concentration of the investigated compounds and their percentage of radical scavenging ability, the compounds’ inhibitory percentages and the concentrations required to block 50% of DPPH radicals (“IC50” values) were determined.

##### Ferric-Reducing Power (FRAP) Assay

The synthesized pyrazole’s antioxidant capacity was assessed by adhering to the procedures previously published in the literature [52]. A UV spectrophotometer was used to measure the absorbances of the reaction mixture at 700 nm, while the target pyrazole compounds, O 1–5, were tested at different doses. BHT served as the standard antioxidant. The mean absorbance of each chemical was determined following three trials. The ferric-reducing antioxidant capacity was determined by measuring the absorbance of the investigated substances at 700 nm.

#### 3.4.2. Inspection of Antibacterial Activity

##### Microbial and Fungal Strains Tested

The antimicrobial activity of new pyrazole derivatives was carried out against two fungal strains (*Candida albicans* ATCC10231 and *Aspergillus niger* MTCC9913) and against four bacterial strains (*Staphylococcus aureus* ATCC6633, *Escherichia coli* K12, *Bacillus subtilis* DSM6333, and *Pseudomonas aeruginosa* CIP82.114), which were provided by the Laboratory of Biotechnology, Environment, Agri-food and Health, Faculty of Sciences Dhar El Mahraz, University Sidi Mohammed Ben Abdellah, Fez, Morocco.

##### Evaluation of Antibacterial and Antifungal Activity

The antibacterial and antifungal activity of the new pyrazole derivatives was assessed by disk diffusion [53]. Briefly, Petri dishes containing Mueller–Hinton (MH) culture medium for bacteria and malt extract for *C. albicans* and *A. niger* were seeded with the four bacterial strains tested and the fungal strains, respectively, by the double-layer method from a fresh culture of each bacterium in liquid MH medium or from a fresh culture of fungal strains in liquid malt extract medium. Decimal dilutions were made in sterile saline (0.9% NaCl) until a turbidity of 0.5 McFarland (10^6^ to 10^8^ CFU/mL) was obtained. Subsequently, 100 µL of each bacterial and fungal culture was added to each tube containing 5 mL of soft agar (0.5% agar–agar in MH medium). The inoculated tubes were poured into Petri dishes containing MH medium and malt extract medium for the bacterial and fungal strains, respectively. Sterile Whatman paper discs (6 mm diameter) were impregnated with 20 μL of new pyrazole derivatives with a concentration of 100 µL in 100 µL of DMSO 5%, then inoculated in Petri dishes with bacteria and yeast (10^6^ to 10^8^ CFU/mL). Positive controls were performed following the same procedures and using the antibiotic streptomycin (30 µg/disk) for the bacteria and the antibiotic Fluconazole (15 mg/mL) for the fungi. Inoculated Petri dishes were incubated at 37 °C and 30 °C, respectively, in the dark and in a humidity-saturated atmosphere. The inhibition diameter was measured after 24 to 48 h of incubation, and in the case of *A. niger* it was recorded after 7 days [54,55].

##### Minimum Inhibitory Concentration (MIC) and Minimum Bactericidal Concentration (MBC) Determination

Determination of the minimum inhibitory concentrations (MICs) and minimum bactericidal concentrations (MBCs) of the new pyrazole derivatives against the four bacterial strains and *C. albicans* was carried out using the microdilution method described by Sarker et al. [56]. In summary, a microdilution was performed by diluting the sample by a factor of 2 in each well, with the exception of the last well, which was used as a positive control for growth. After 24 h incubation for bacteria and 48 h for *C. albicans* and 7 days for *A. niger*, the MICs and BMCs were determined using the colorimetric method (TTC 0.2% (*w*/*v*)) [57].

##### Statistical Analysis

The results were expressed as means of triplicate experiments (standard deviations). Analysis of variance was used to determine the significance of the difference between means (two-way ANOVA). GraphPad Prism was used to perform Tukey’s multiple-range tests at α = 0.05 [58].

### 3.5. Drug-likeness Properties and Physicochemical Features

The study involved the assessment of drug-likeness and qualitative evaluation to ascertain the potential of a molecule to serve as an orally administered drug with favorable bioavailability [59]. Five compounds underwent scrutiny for physicochemical properties, drug-likeness, and bioavailability parameters, utilizing the Swiss-ADME online tool (http://swissadme.ch/, accessed on 15 November 2024) [60] and Molinspiration’s online physicochemical property calculation software (https://www.molinspiration.com/cgi-bin/properties, accessed on 15 November 2024). Additionally with the parameters assessed including the partition coefficient (log P), molar refractivity, molecular weight, number of heavy atoms, number of hydrogen donors, number of hydrogen acceptors, and number of violations, further evaluation of drug-likeness parameters, encompassing Pfizer Rule 3/75, GSK Rule 4/400, and Pfizer’s Golden Triangle, was executed through the ADMETlab v2.0 online server (https://admetmesh.scbdd.com/, accessed on 15 November 2024) [61].

### 3.6. In Silico ADMET Profile Prediction

Based on the ideas of Lipinski [62], which outline the molecular characteristics that are important for evaluating important pharmacokinetic characteristics, such as absorption, distribution, metabolism, excretion, and toxicity, the ADMET approach is essential for creating and advancing new drug candidate molecules [63]. The online program pkCSM (http://biosig.unimelb.edu.au/pkcsm/prediction, accessed on 17 November 2024) [64] was used in the current study. In order to predict ADMET characteristics, the candidate molecular structures that were chosen were uploaded in SMILES format. These structures were then used to predict the following: distribution (permeability of the blood–brain barrier, the unbound fraction, permeability of the central nervous system, and the steady-state volume of distribution), metabolism (CYP2D6/CYP3A4 substrate and cytochrome P450 inhibitors), excretion (total drug clearance), and toxicity (skin sensitization, hepatotoxicity, and hERG I and II inhibitors) [65].

### 3.7. Molecular Docking Analysis

Molecular docking studies were realized using the AutoDock Vina 1.5.6 software tool [66]. This tool was utilized to assess the potential docking modes of interaction between the ligand and relevant biological sites. CATALASE COMPOUND II (PDB ID: 2CAG) was employed to elucidate the mechanism of antioxidant activity, and the crystal structure of E. coli Topoisomerase IV co-complexed with an inhibitor (PDB ID: 3FV5) was utilized to investigate the mechanism of antibacterial activity. Also, a 3D crystal structure of Cytochrome P450 14α-sterol demethylase (CYP51) from *Mycobacterium tuberculosis* complexed with fluconazole (PDB ID: 1EA1) was analyzed to understand the enzyme’s mechanism of antifungal activity. These three proteins were obtained in PDB format from the RCSB Protein Data Bank (www.rcsb.org, accessed on 20 November 2024), and the reference drug was acquired from the Zinc Database [67].

In order to optimize the proteins, it was necessary to delete the water molecules and existing complex ligands, after which Kollman and Geister charges, including polar hydrogen, were added. The chemical compound structures (ligands) were drawn, optimized, and saved in mol2 format, which was then converted into PDBQT format.

For identifying the binding sites, grids were generated all around the active site of each protein, employing the following central coordinates: x = 12.51, y = −1.13, and z = 3.26 for E. coli Topoisomerase IV and x = 59.83, y = 14.86, and z = 16.24 for CATALASE COMPOUND II, and in the case of Cytochrome P450 14α-sterol demethylase (CYP51) from Mycobacterium tuberculosis, the central coordinates were x = −18.14, y = −6.64, and z = 66.54. The grid size was set to X = 20, Y = 20, and Z = 20, with a spacing of 0.375 Å. Ligand-binding affinity, measured in kcal/mol units, was scored as a negative classification. Using Biovia Discovery Studio 2019 and PyMol (The PyMOL Molecular Graphics System, Version 2.0 Schrödinger, LLC), the interactions between proteins and ligands were visualized in both 2D and 3D and thoroughly examined.

## 4. Conclusions

This study successfully synthesized and characterized novel mono- and bis-pyrazole derivatives using an environmentally friendly, catalyst-free method. Theoretical analyses using DFT supported the experimental findings, demonstrating the stability and reactivity of the compounds. Among the synthesized derivatives, O4 emerged as the most promising candidate, exhibiting potent antioxidant activity with significant DPPH radical scavenging and IC50 values, alongside exceptional antimicrobial efficacy against bacterial and fungal strains, including *Pseudomonas aeruginosa* and *Candida albicans*. Computational studies confirmed favorable pharmacokinetics, the absence of AMES toxicity, and strong binding affinities, further validating the therapeutic potential of O4. These results highlight the efficacy of combining green chemistry and computational approaches in drug discovery, paving the way for the development of pyrazole-based therapeutic agents to address challenges such as antimicrobial resistance and oxidative stress. Future research will include the performance of molecular dynamics simulations to confirm the stability of the docking results and further refine the SAR for more targeted activity optimization.

## Figures and Tables

**Table 1:** The electronic FMO energies and global reactivity descriptors of the compounds R1, R2, R3, R4, and R5.

Molecule	HOMO (eV)	LUMO (eV)	*μ* (eV)	*N*	η (eV)	ω	E Gap I	E Gap II
R1	−6.437	2.214	−2.111	2.312	8.651	0.258	----	----
R2	−5.662	2.286	−1.688	3.087	7.948	0.179	−7.876	−8.723
R3	−6.422	2.216	−2.103	2.327	8.638	0.256	−8.636	−8.653
R4	−5.986	2.317	−1.834	2.763	8.303	0.203	−8.200	−8.754
R5	−6.038	2.304	−1.867	2.711	8.342	0.209	−8.252	−8.741

**Table 2:** Capabilities of reducing power of synthesized mono- and bis-pyrazoles and standard antioxidant (BHT).

	O1	O2	O3	O4	O5	BHT
**Concentrations (µg/mL)**			%			
**50**	0.4690 ± 0.0343	0.1782 ± 0.0764	0.1090 ± 0.0604	0.1840 ± 0.0507	0.2030 ± 0.0184	0.1290 ± 0.0184
**150**	0.5810 ± 0.0312	0.2880 ± 0.0702	0.2490 ± 0.0509	0.4580 ± 0.0587	0.3070 ± 0.0085	0.4210 ± 0.0778
**300**	0.6510 ± 0.0678	0.3400 ± 0.0225	0.3840 ± 0.0203	0.5790 ± 0.0204	0.4280 ± 0.0292	0.5430 ± 0.0453
**600**	0.6920 ± 0.0385	0.4420 ± 0.0283	0.4910 ± 0.0517	0.6170 ± 0.0172	0.5670 ± 0.0283	0.6340 ± 0.0337

Values are represented as means ± standard deviations of triplicate experiments.

**Table 3:** Free radical scavenging percentage measured for synthesized mono- and bis-pyrazoles and standard antioxidant (BHT).

	O1	O2	O3	O4	O5	BHT
**Concentrations (µg/mL)**			%			
62.5	55.80 ± 2.92	63.62 ± 4.19	57.54 ± 3.71	65.36 ± 3.42	62.17 ± 2.44	73.19 ± 2.44
125	57.39 ± 2.23	62.90 ± 4.01	60.72 ± 3.42	66.96 ± 3.66	65.80 ± 2.15	77.97 ± 4.23
250	59.86 ± 3.93	63.19 ± 2.57	67.68 ± 2.50	75.22 ± 2.50	67.97 ± 2.77	81.45 ± 3.25
500	60.14 ± 3.05	63.33 ± 2.74	69.71 ± 3.45	80.14 ± 2.41	68.84 ± 2.74	82.75 ± 2.91
1000	61.74 ± 3.10	73.77 ± 3.83	67.68 ± 2.54	80.72 ± 3.23	72.90 ± 3.40	83.04 ± 2.20

Values are represented as means ± standard deviations of triplicate experiments.

**Table 4:** EC50 values calculated for both DPPH and FRAP methods of O1–5 and butylated hydroxytoluene.

	BHT	O1	O2	O3	O4	O5
DPPH EC-50 (µg/mL)	22.98 ± 0.46	20.62 ± 0.46	171.85 ± 3.70	66.84 ± 2.55	40.91 ± 0.68	50.64 ± 3.06
FRAP EC-50(µg/mL)	362.04 ± 6.16	115.84 ± 2.25	978.62 ± 19.6	920.59 ± 27.94	289.11 ± 15.4	572.84 ± 23.78

Values are represented as means ± standard deviations of triplicate experiments.

**Table 5:** MICs and MBCs of new pyrazole derivatives with standard Streptomycine and Fluconazole against bacterial and fungal strains expressed in mg/mL.

		*P. aeruginosa* CIP82.114	*S. aureus* ATCC6633	*E. coli* K12	*B. subtilis* DSM6333	*C. albicans* ATCC10231	*Aspergillus niger* MTCC9913
O1	MIC	0.191 ± 0.020 ^a^	0.048 ± 0.004 ^a^	0.024 ± 0.006 ^a^	0.024 ± 0.002 ^a^	0.048 ± 0.005 ^a^	0.024 ± 0.006 ^a^
	MBC	0.191 ± 0.033 ^A^	0.048 ± 0.004 ^A^	0.191 ± 0.005 ^A^	0.048 ± 0.011 ^A^	0.048 ± 0.007 ^A^	0.024 ± 0.003 ^A^
O2	MIC	0.237 ± 0.028 ^b^	0.059 ± 0.005 ^a^	0.059 ± 0.010 ^b^	0.059 ± 0.006 ^b^	0.118 ± 0.014 ^b^	0.059 ± 0.012 ^b^
	MBC	0.237 ± 0.025 ^B^	0.059 ± 0.006 ^A^	0.059 ± 0.007 ^B^	0.059 ± 0.012 ^A^	0.118 ± 0.012 ^B^	0.059 ± 0.015 ^B^
O3	MIC	0.133 ± 0.015 ^c^	0.033 ± 0.005 ^c^	0.033 ± 0.006 ^c^	0.033 ± 0.007 ^c^	0.067 ± 0.010 ^b^	0.008 ± 0.002
	MBC	0.267 ± 0.015 ^D^	0.033 ± 0.004 ^C^	0.033 ± 0.008 ^C^	0.033 ± 0.005 ^C^	0.067 ± 0.008 ^B^	0.008 ± 0.001
O4	MIC	0.129 ± 0.013 ^c^	0.032 ± 0.006 ^c^	0.016 ± 0.004 ^b^	0.016 ± 0.003 ^a^	0.032 ± 0.006 ^c^	0.016 ± 0.004 ^b^
	MBC	0.129 ± 0.031 ^C^	0.032 ± 0.003 ^C^	0.032 ± 0.003 ^B^	0.032 ± 0.005 ^A^	0.032 ± 0.009 ^C^	0.016 ± 0.002 ^B^
O5	MIC	0.140 ± 0.025 ^c^	0.017 ± 0.004 ^a^	0.035 ± 0.008 ^b^	0.017 ± 0.002 ^b^	0.035 ± 0.008 ^a^	0.009 ± 0.004 ^b^
	MBC	0.280 ± 0.028 ^D^	0.035 ± 0.005 ^A^	0.035 ± 0.006 ^B^	0.035 ± 0.003 ^B^	0.035 ± 0.007 ^A^	0.009 ± 0.002 ^B^
Streptomycine	MIC	0.243 ± 0.012 ^a^	1.560 ± 0.045 ^b^	3.125 ± 0.012 ^a^	7.125 ± 0.046 ^c^	-	-
	MBC	0.243 ± 0.034 ^A^	1.560 ± 0.028 ^B^	3.125 ± 0.009 ^A^	7.125 ± 0.025 ^C^	-	-
Fluconazole	MIC	-	-	-	-	0.0156 ± 0.005	7.1250 ± 0.004
	MBC	-	-	-	-	0.0156 ± 0.019	7.1250 ± 0.002

Mean values (±SDs, *n* = 3) followed by different small letters (for MICs) and capital letters (for MBCs) in the same column are significantly different (two-way ANOVA; Tukey test, *p* < 0.05).

**Table 6:** Physicochemical properties of the synthesized pyrazole derivatives determined by Molinspiration.

Compound	MW	TPSA	N-Atoms	Volume	nON	nOHNH	Nviolations	Nrotb	milogP
O1	374.58	42.13	27	393.81	6	0	0	11	3
O2	266.43	33.09	19	290.88	4	1	0	9	2.25
O3	275.40	38.89	20	280.45	5	0	0	5	2.02
O4	353.47	48.12	26	344.26	6	0	0	7	2.78
O5	337.47	38.89	25	335.52	5	0	0	7	3.16

**Table 7:** Drug-likeness properties of the synthesized pyrazole-derivative compounds determined by Swiss ADME ^1^ and AdmetLab 2.0 ^2^.

Compound	Drug-likeness							
	Veber ^1^	Muegge ^1^	Egan ^1^	Ghose ^1^	Lipinski ^1^	Pfizer Rule ^2^	GSK Rule ^2^	Golden Triangle ^2^
O1	No	Yes	Yes	Yes	Yes	Accepted	Accepted	Accepted
O2	Yes	Yes	Yes	Yes	Yes	Accepted	Accepted	Accepted
O3	Yes	Yes	Yes	Yes	Yes	Accepted	Accepted	Accepted
O4	Yes	Yes	Yes	Yes	Yes	Accepted	Accepted	Accepted
O5	Yes	Yes	Yes	Yes	Yes	Accepted	Accepted	Accepted

**Table 8:** Computational ADMET results of pyrazole derivatives O 1–5.

		O1	O2	O3	O4	O5	Unit
***Absorption***							
**Intestinal absorption (human)**		97.46	92.945	95.017	97.389	95.77	Numeric (% absorbed)
**Distribution**							
**VDss (human)**		1.294	1.156	0.569	0.735	0.688	Numeric (log L/kg)
**BBB permeability**		−0.042	0.137	0.109	−0.044	0.192	Numeric (log BB)
**CNS permeability**		−4.031	−3.457	−3.309	−1.891	−1.736	Numeric (log PS)
***Metabolism***							
**CYP**	2D6 substrate	No	No	No	No	No	Categorical (yes/no)
	3A4 substrate	Yes	No	No	Yes	Yes	Categorical (yes/no)
	2C19 inhibitor	No	No	No	No	No	Categorical (yes/no)
	2C9 inhibitor	No	No	No	Yes	No	Categorical (yes/no)
	1A2 inhibitor	No	No	No	Yes	Yes	Categorical (yes/no)
	3A4 inhibitor	No	No	No	No	No	Categorical (yes/no)
	2D6 inhibitor	Yes	No	Yes	No	Yes	Categorical (yes/no)
***Excretion***							
	Total clearance	1.035	1.161	0.886	0.885	0.971	Numeric (log mL/min/kg)
***Toxicity***							
	AMES toxicity	No	No	No	No	No	Categorical (yes/no)

**Table 9:** Biding energy of the pyrazole derivatives **(O1–5)** and BHT with **2CAG** determined using auto dock vina.

Ligand	Binding Energy (kcal/mol)	Ligand–Protein Key Amino Acid Interactions	Predominant Type of Interaction	Number of Conventional Hydrogen Bonds
O1	−7.6	ARG52 SER336 PHE140 ARG333 TYR337	Hydrophobic	3
O2	−6.5	TYR337 VAL125 ALA112 HIS54	Hydrophobic	0
O3	−8.1	PHE140 TYR337 ALA340 HIS54 ALA112 VAL125	Hydrophobic	0
O4	−8.4	ARG52 ALA340 ARG51 VAL125 ALA112 ARG91 SER93 ARG333 PHE140 TYR337	Hydrophobic	1
O5	−8.7	ARG333 PHE140 TYR337 ARG52 ARG344 ALA340 ARG51 ALA112 VAL125 HIS54	Hydrophobic	0
BHT	−7.3	VAL125 HIS341 ALA340 ARG51 PHE140 TYR337 HIS54	Hydrophobic	0

**Table 10:** Biding energy of the pyrazole derivatives (O1–5) and streptomycin with 3FV5 determined using auto dock vina.

Ligand	Binding Energy (kcal/mol)	Ligand–Protein Key Amino Acid Interactions	Predominant Type of Interaction	Number of Conventional Hydrogen Bonds
O1	−5.0	ARG72 PRO75 MET74 ALA86 ILE90	Hydrophobic	0
O2	−4.3	ASN42 MET74 ILE90 GLU46 ARG72	Hydrophobic	1
O3	−5.3	ALA49 ARG72 GLU46 MET74 ASN42 SER43	Hydrophobic	0
O4	−6.0	PRO75 MET74 ASP69 ASN42 GLU46 ALA49 ARG72	Hydrophobic	0
O5	−5.3	ALA49 ASP45 MET74 ASN42	Hydrophobic	0
Streptomicyne	−5.4	HIS51 ARG72 ASN42	Hydrogen bonds	3

**Table 11:** Biding energy of the pyrazole derivatives (O1–5) and Fluconazole with 1EA1 determined using auto dock vina.

Ligand	Binding Energy (kcal/mol)	Ligand–Protein Key Amino Acid Interactions	Predominant Type of Interaction	Number of Conventional Hydrogen Bonds
O1	−6.4	LEU321 TYR76 PHE78 ARG96 ALA256 LEU100 CYS394	Hydrophobic	1
O2	−5.6	TYR76 CYS394 LEU324 LEU321 PHE78 HIS259 VAL434	Hydrophobic	0
O3	−6.7	ALA256 ARG96 LEU100 PHE83 LEU321 MET79 PHE78 TYR76	Hydrophobic	0
O4	−8.5	TYR76 PHE78 MET79 ILE323 LEU321 MET433 CYS394 LEU324	Hydrophobic	0
O5	−8.4	ARG96 LEU100 ALA256 PHE78 LEU321 VAL434	Hydrophobic	1
Fluconazole	−7.0	SER507 MET508 HIS377 ALA61 LEU88 LEU87 TYR64 PHE233 PRO230	Hydrophobic	1

## Data Availability

The original contributions presented in this study are included in the article/Supplementary Material. Further inquiries can be directed to the corresponding author.

## Supplementary Materials

The following supporting information can be downloaded at: https://www.mdpi.com/article/10.3390/ph18020167/s1, Figure S1: The 1H-NMR spectrum of the compounds O1; Figure S2: The 13C-NMR spectrum of the compounds O1; Figure S3: The Masse spectrum of the compounds O1; Figure S4: The 1H-NMR spectrum of the compounds O2; Figure S5: The 13C-NMR spectrum of the compounds O2; Figure S6: The masse spectrum of the compounds O2; Figure S7: The 1H-NMR spectrum of the compounds O3; FigureS8: The 13C-NMR spectrum of the compounds O3; Figure S9: The masse spectrum of the compounds O3; Figure S10: The 1H-NMR spectrum of the compounds O4; Figure S11: The 13C-NMR spectrum of the compounds O4; Figure S12: The masse spectrum of the compounds O4; Figure S13: The 1H-NMR spectrum of the compounds O5; Figure S14: The 13C-NMR spectrum of the compounds O5; Figure S15: The 1H-NMR spectrum of the compounds O5.
