# Supplementary material for: Environmentally Friendly Synthesis of New Mono- and Bis-Pyrazole Derivatives; In Vitro Antimicrobial, Antifungal, and Antioxidant Activity; and In Silico Studies: DFT, ADMETox, and Molecular Docking
Source: Pharmaceuticals (Basel). 2025 Jan 26;18(2):167. doi: 10.3390/ph18020167 (PMC11858278; doi:10.3390/ph18020167)
Supplement: Supplementary file 1 [file pharmaceuticals-18-00167-s001.zip › pharmaceuticals-3428464-supplementary.pdf]

Environmentally Friendly Synthesis of New Mono- and
Bis-Pyrazole Derivatives; In Vitro Antimicrobial, Antifungal, and Antioxidant Activity; and In Silico Studies: DFT, ADMETox, and Molecular Docking

Oussama Merzouki [1,*], Nadia Arrousse [2], Elhachmia Ech-chihbi [3], Ashwag S. Alanazi [4], El Houssine Mabrouk [1,5], Mohamed Hefnawy [6], Abdelfattah El Moussaoui [7,8], Hanane Touijer [8], Azeddin El Barnossi [8] and Mustapha Taleb [1]

[1] Laboratory of Engineering Electrochemistry, Modeling, and Environment, Department of Chemistry,
Faculty of Sciences Dhar Mahraz, Sidi Mohamed Ben Abdellah University, Fez 30000, Morocco
[2] School of Science and Engineering, Al Akhawayn University in Ifrane, Hassan II Avenue,
Ifrane 53000, Morocco
[3] Laboratory of Physics and Chemistry of Inorganic and Organic Materials, Higher Normal School,
Mohammed V University, Rabat 30050, Morocco
[4] Department of Pharmaceutical Sciences, College of Pharmacy, Princess Nourah Bint Abdulrahman
University, Riyadh 11671, Saudi Arabia
[5] Laboratory of Materials Engineering for the Environment and Natural Ressources, Faculty of Sciences and Technics, University of Moulay Ismail, Meknes, B.P 509, Boutalamine, Errachidia 52000, Morocco
[6] Department of Pharmaceutical Chemistry, College of Pharmacy, King Saud University, Riyadh 11451, Saudi Arabia
[7] Plant Biotechnology Team, Faculty of Sciences, Abdelmalek Essaadi University, Tetouan 93002, Morocco
[8] Laboratory of Biotechnology, Environment, Agrifood, and Health, Faculty of Sciences Dhar El Mahraz,
Sidi Mohamed Ben Abdellah University, Fez 30050, Morocco
* Correspondence: oussama.merzouki@usmba.ac.ma

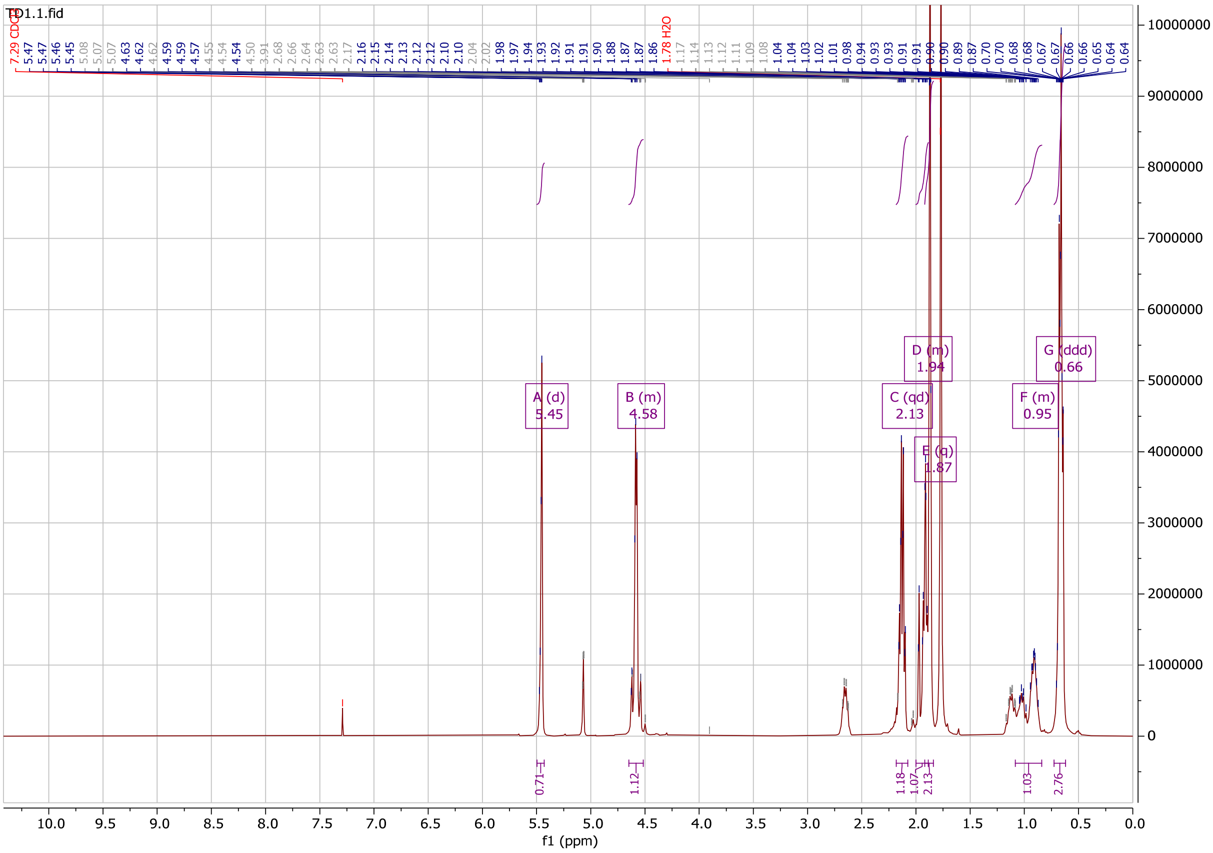

Figure S1. The ^{1}H-NMR spectrum of the compounds **O1**.

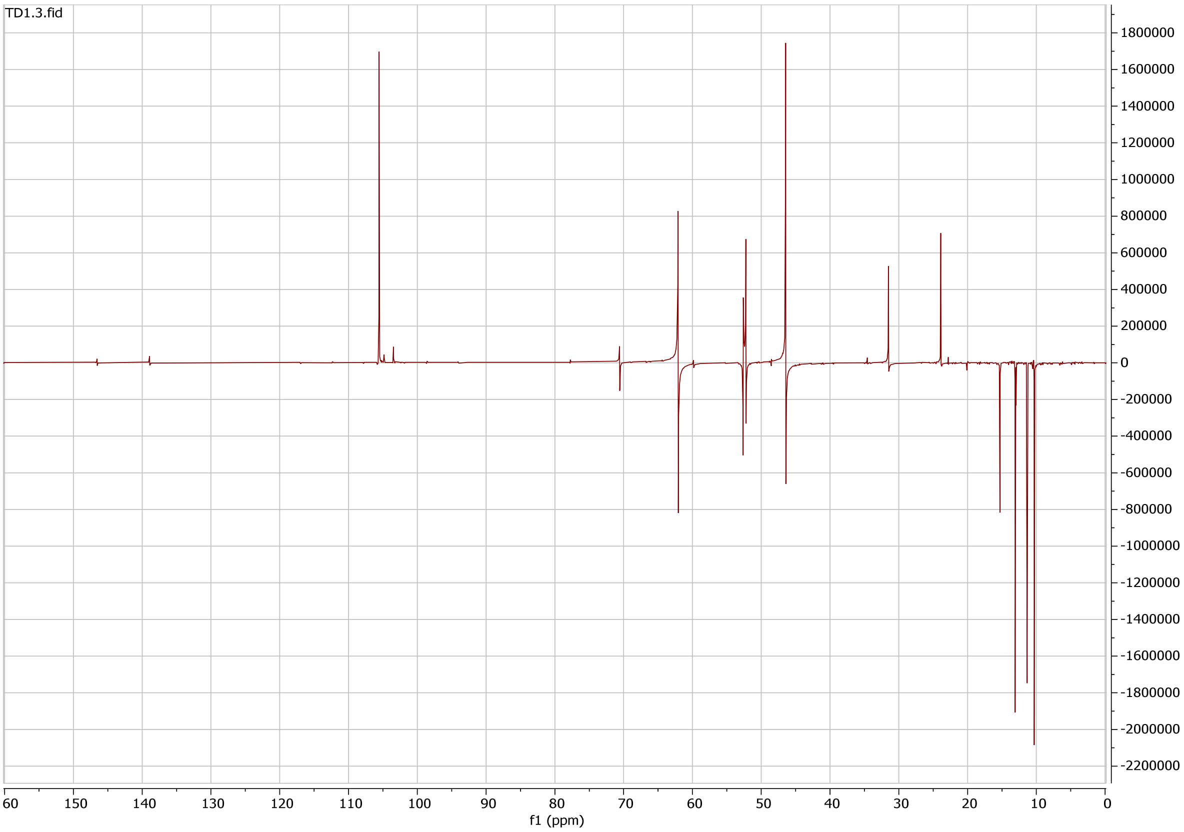

Figure S2. The ^{13}C-NMR spectrum of the compounds **O1**.

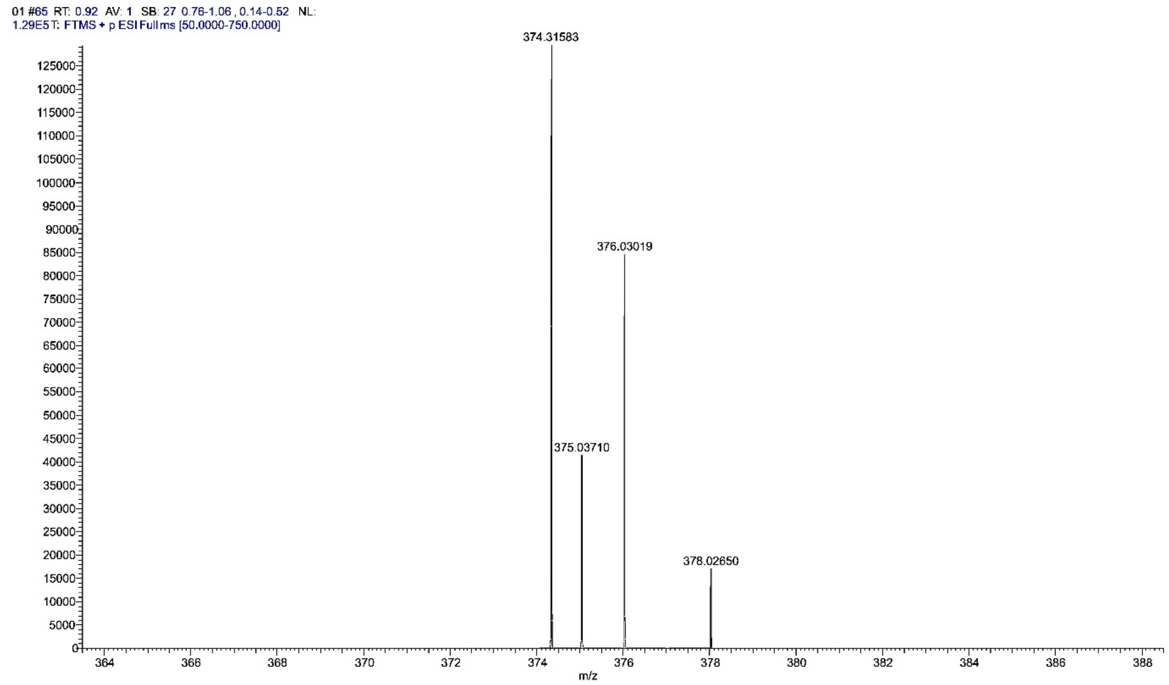

Figure S3. The Masse spectrum of the compounds **O1**.

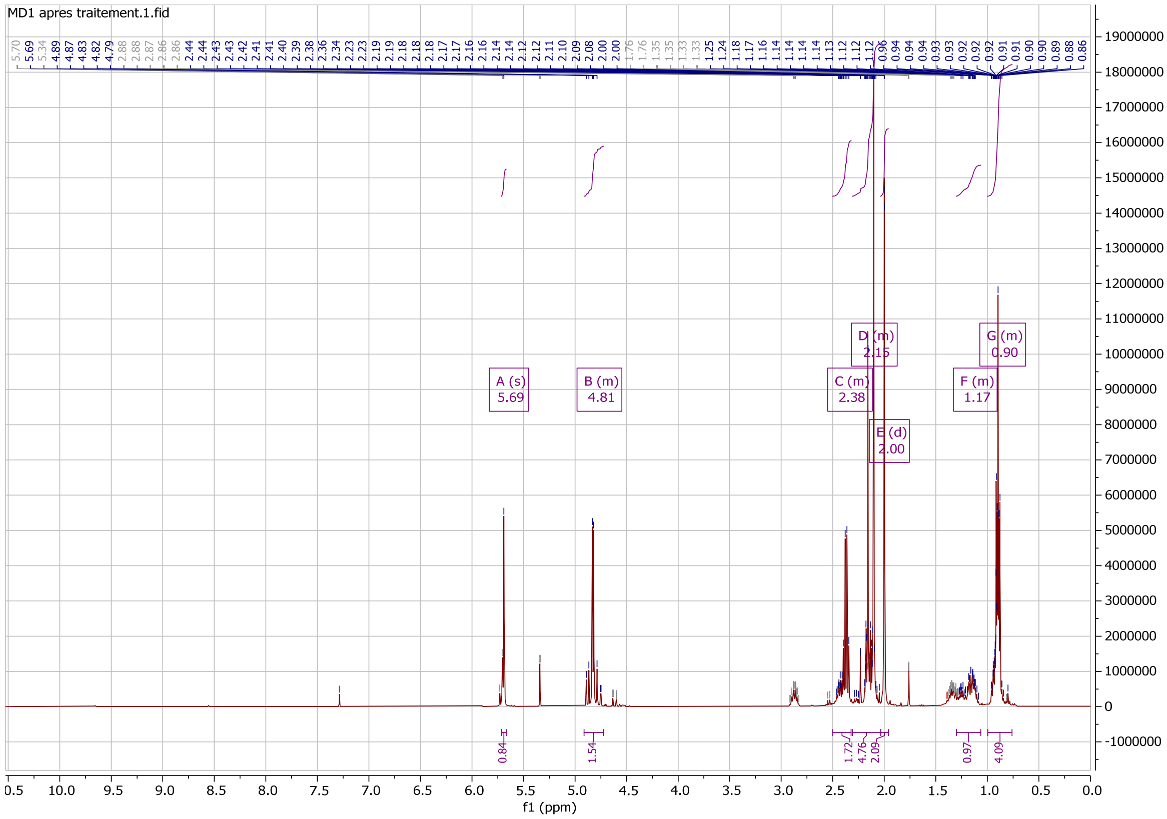

Figure S4. The ^{1}H-NMR spectrum of the compounds **O2**.

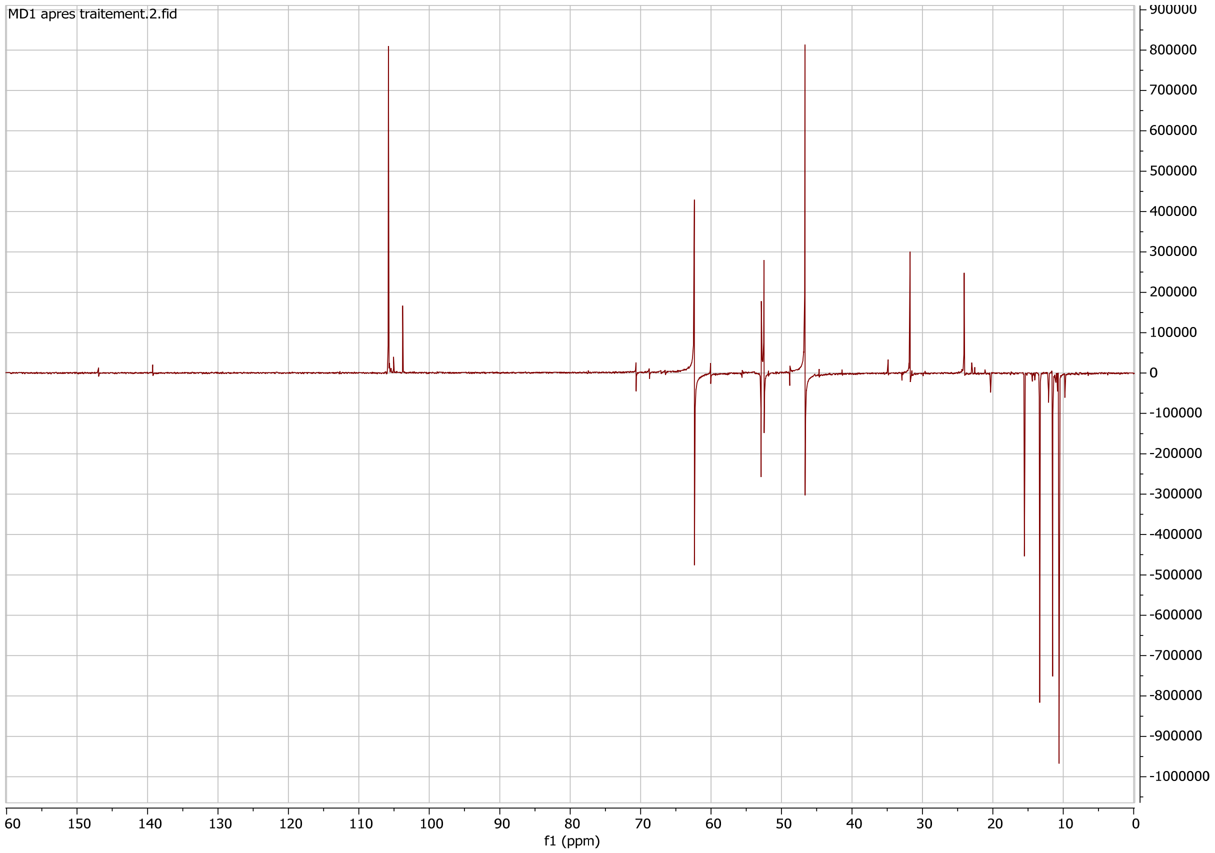

Figure S5. The ^{13}C-NMR spectrum of the compounds **O2**.

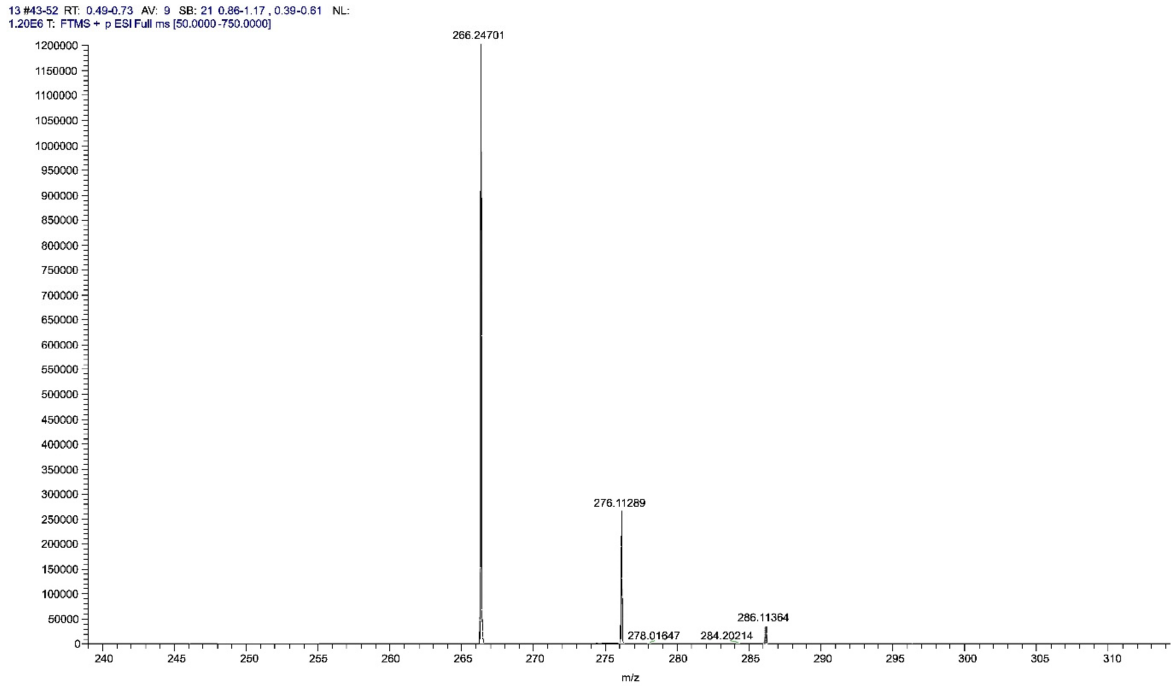

Figure S6. The masse spectrum of the compounds **O2**.

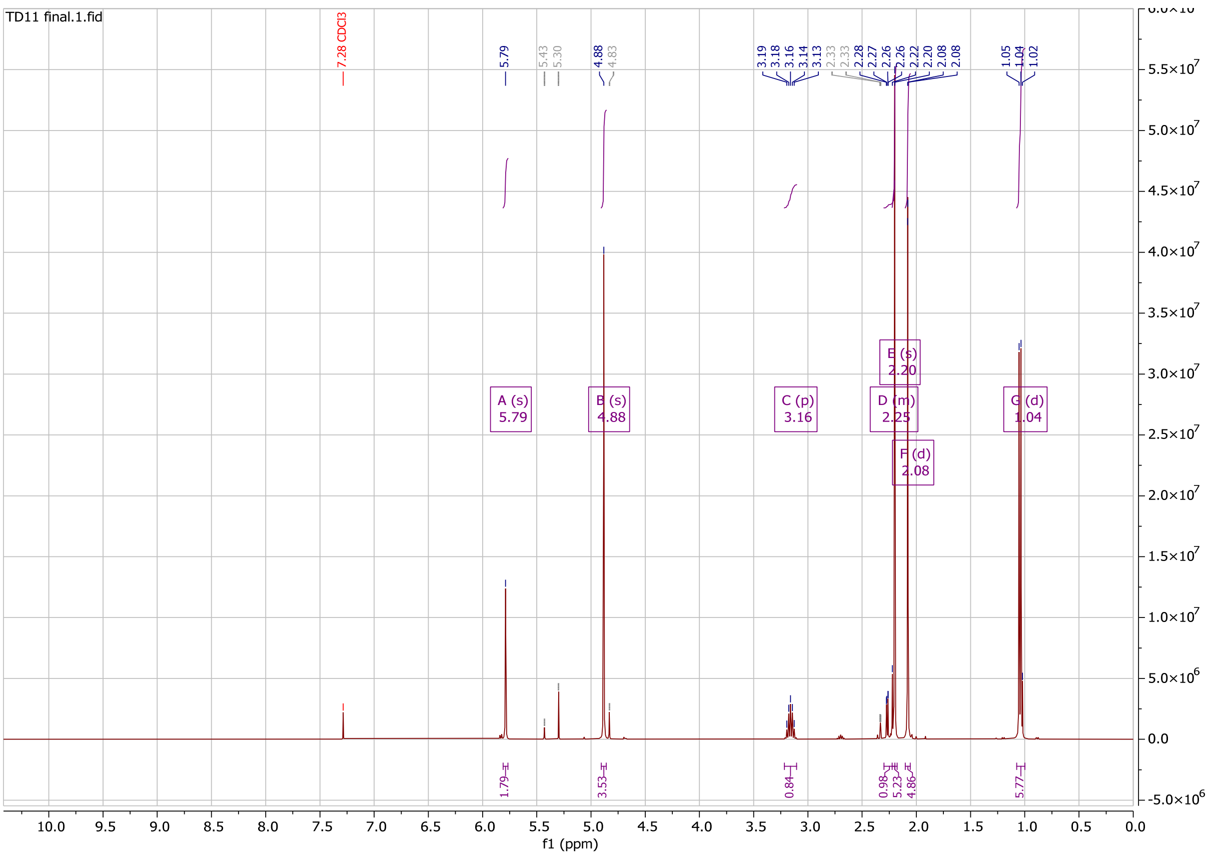

Figure S7. The ^{1}H-NMR spectrum of the compounds **O3**.

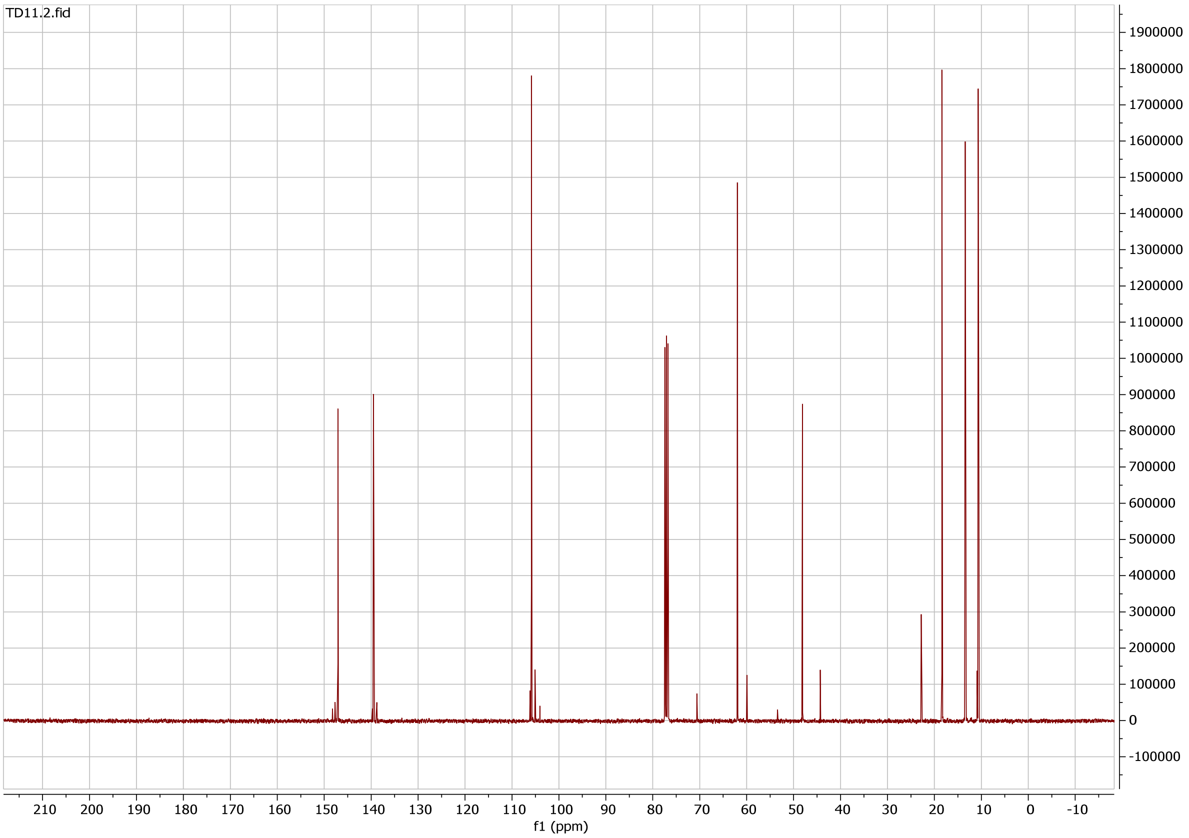

FigureS8. The ^{13}C-NMR spectrum of the compounds **O3**.

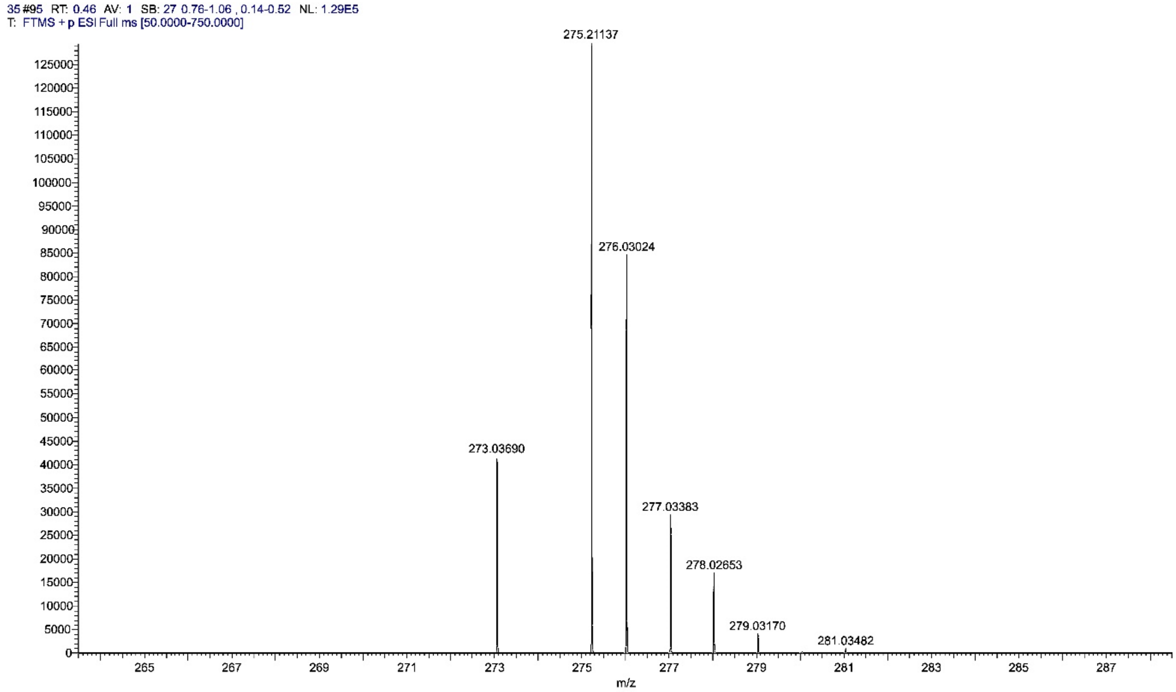

Figure S9. The masse spectrum of the compounds **O3**.

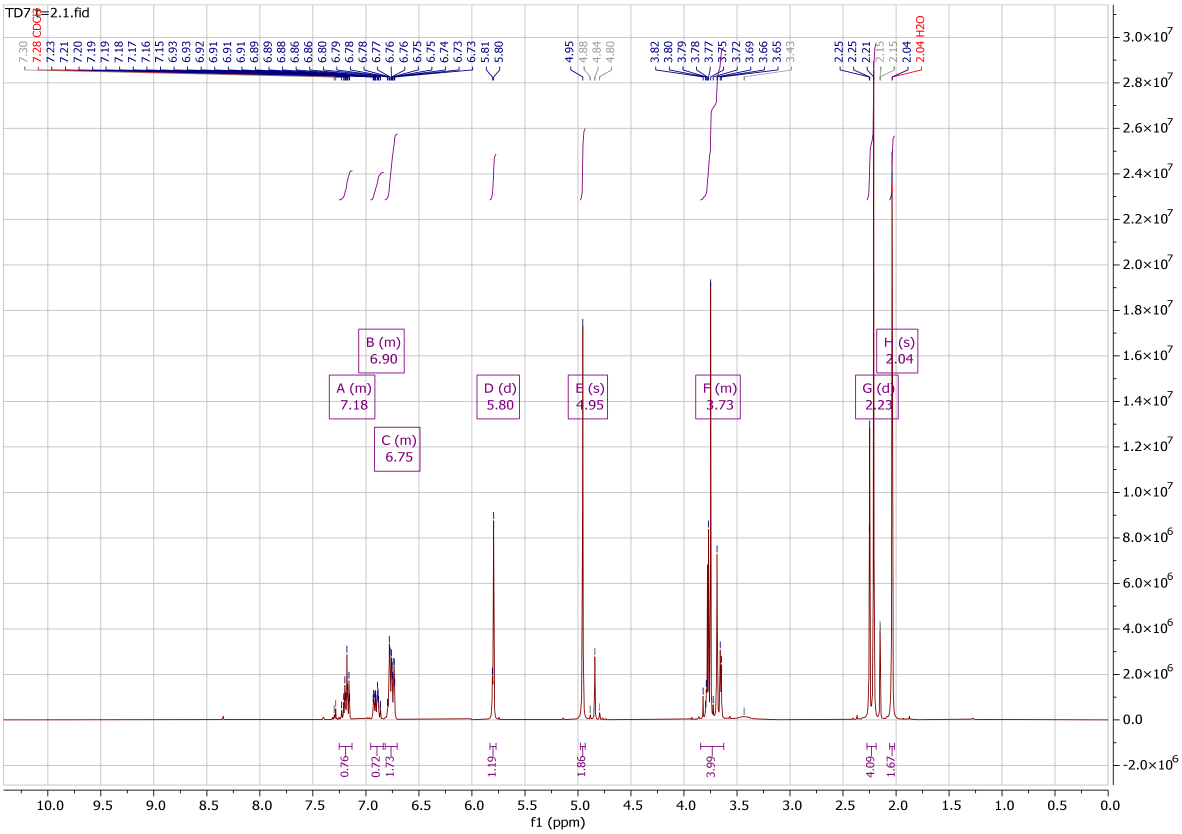

Figure S10. The ^{1}H-NMR spectrum of the compounds **O4**.

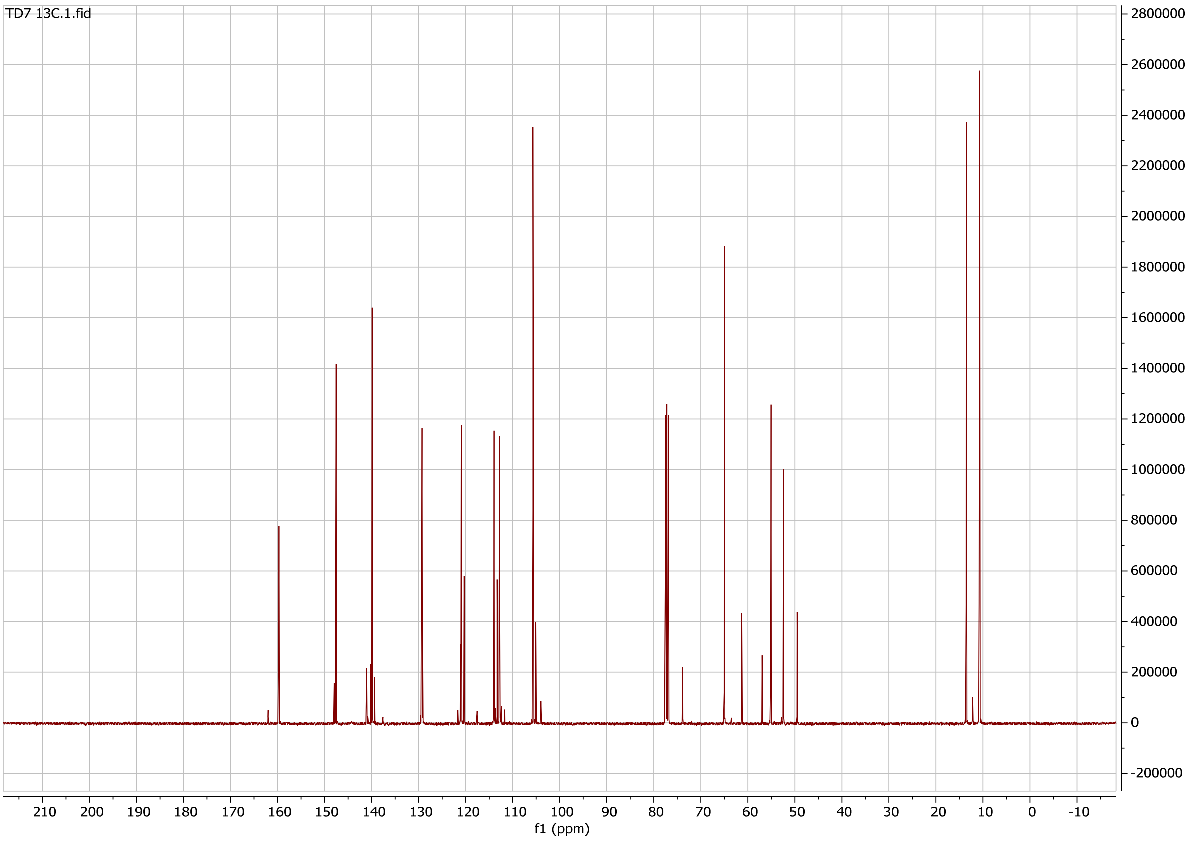

Figure S11. The ^{13}C-NMR spectrum of the compounds **O4**.

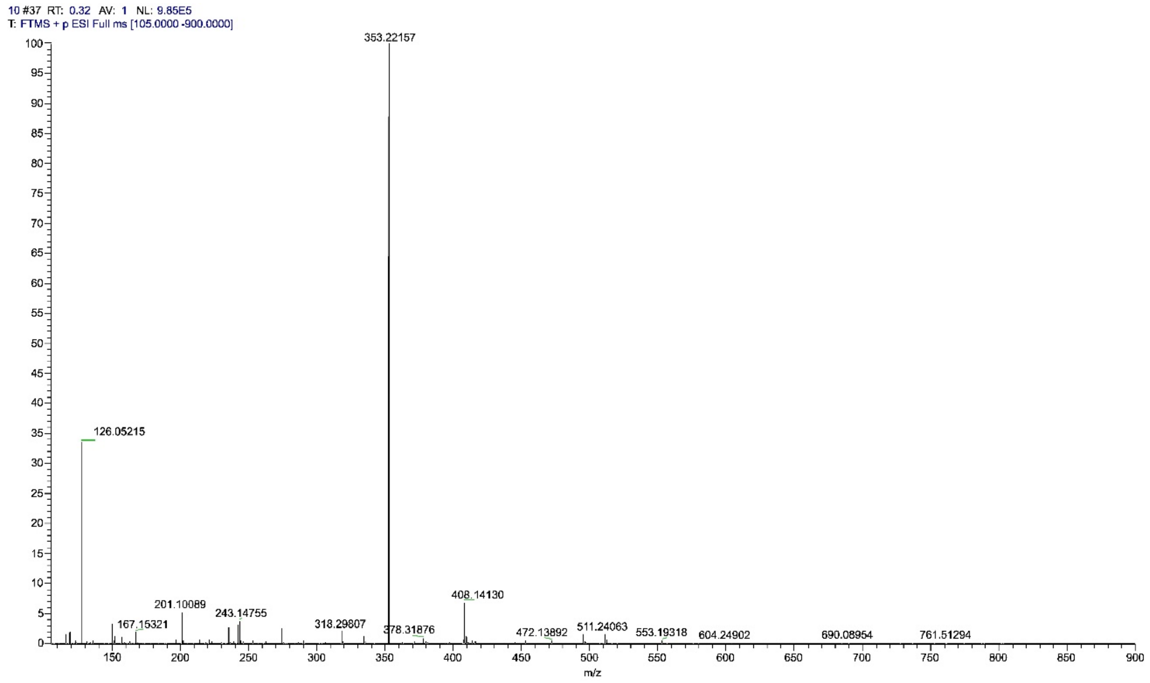

Figure S12. The masse spectrum of the compounds **O4**.

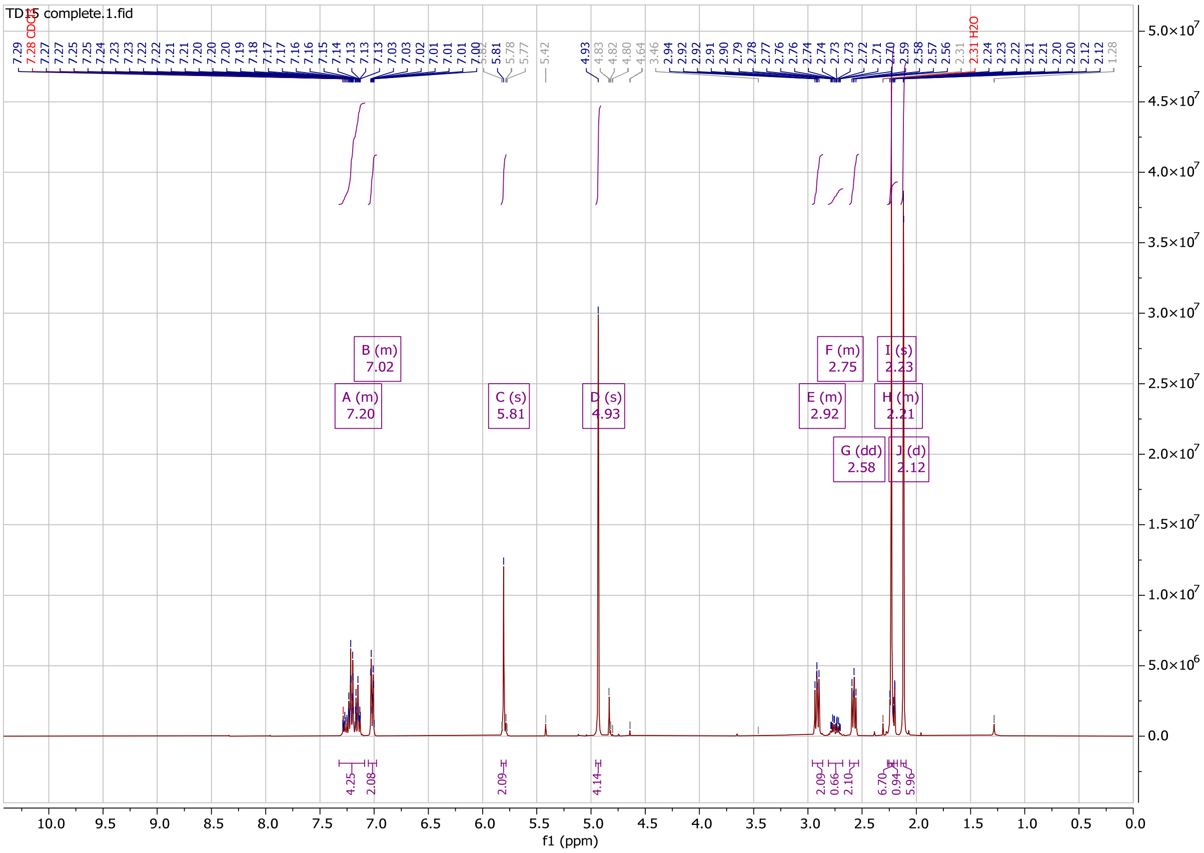

Figure S13. The ¹H-NMR spectrum of the compounds **O5**.

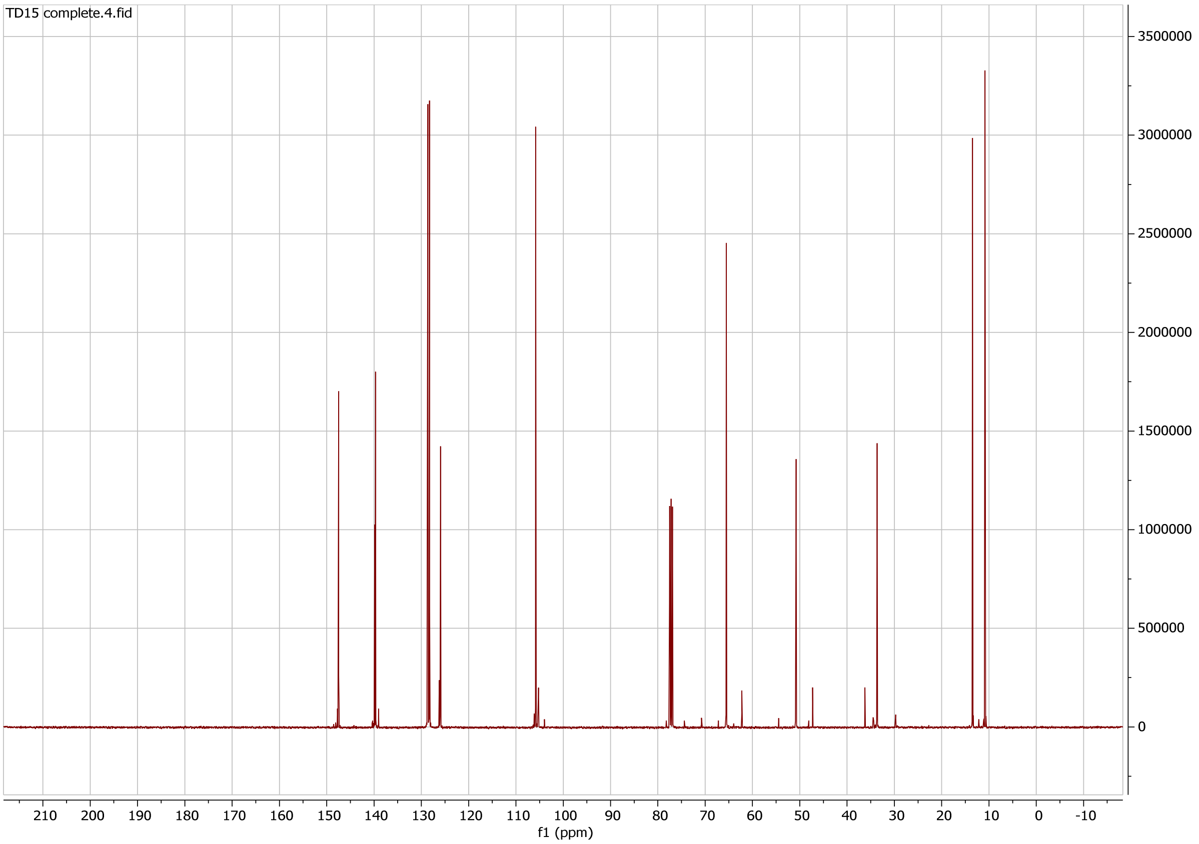

Figure S14. The ^{13}C-NMR spectrum of the compounds **O5**.

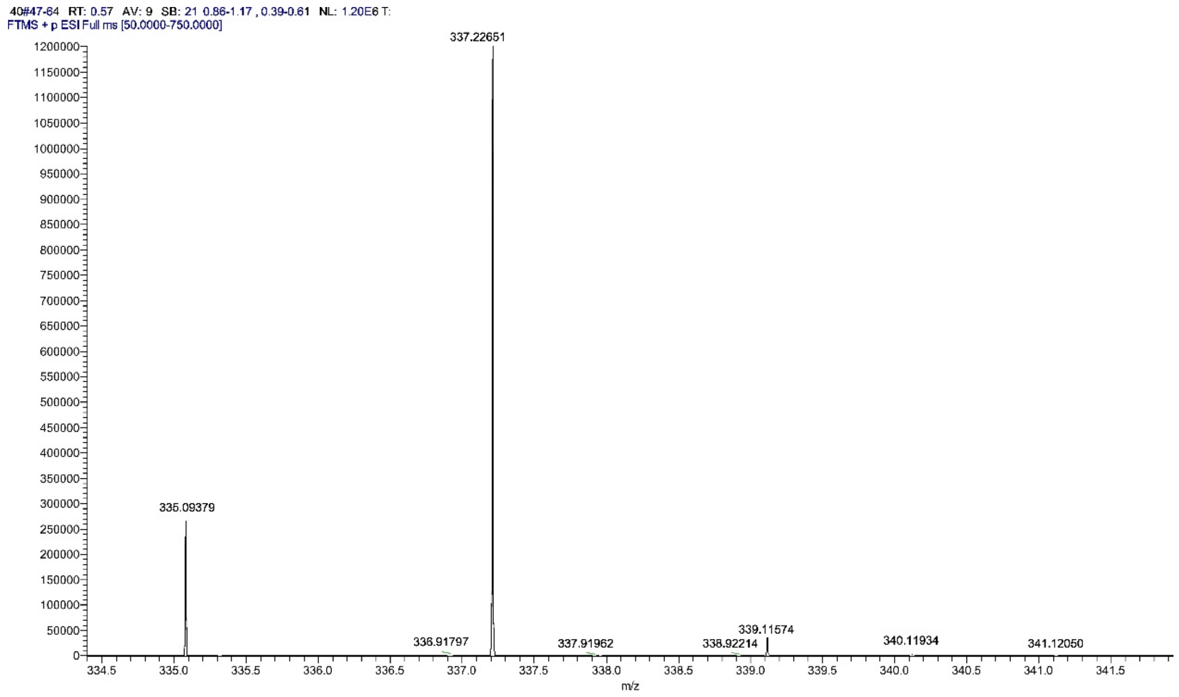

Figure S15. The ^{1}H-NMR spectrum of the compounds **O5**.
